# Direct Ubiquitin Independent Recognition and Degradation of a Folded Protein by the Eukaryotic Proteasomes-Origin of Intrinsic Degradation Signals

**DOI:** 10.1371/journal.pone.0034864

**Published:** 2012-04-10

**Authors:** Amit Kumar Singh Gautam, Satish Balakrishnan, Prasanna Venkatraman

**Affiliations:** Advanced Centre for Treatment, Research and Education in Cancer, Kharghar, Navi Mumbai, India; St. Georges University of London, United Kingdom

## Abstract

Eukaryotic 26S proteasomes are structurally organized to recognize, unfold and degrade globular proteins. However, all existing model substrates of the 26S proteasome in addition to ubiquitin or adaptor proteins require unstructured regions in the form of fusion tags for efficient degradation. We report for the first time that purified 26S proteasome can directly recognize and degrade apomyoglobin, a globular protein, in the absence of ubiquitin, extrinsic degradation tags or adaptor proteins. Despite a high affinity interaction, absence of a ligand and presence of only helices/loops that follow the degradation signal, apomyoglobin is degraded slowly by the proteasome. A short floppy F-helix exposed upon ligand removal and in conformational equilibrium with a disordered structure is mandatory for recognition and initiation of degradation. Holomyoglobin, in which the helix is buried, is neither recognized nor degraded. Exposure of the floppy F-helix seems to sensitize the proteasome and primes the substrate for degradation. Using peptide panning and competition experiments we speculate that initial encounters through the floppy helix and additional strong interactions with N-terminal helices anchors apomyoglobin to the proteasome. Stabilizing helical structure in the floppy F-helix slows down degradation. Destabilization of adjacent helices accelerates degradation. Unfolding seems to follow the mechanism of helix unraveling rather than global unfolding. Our findings while confirming the requirement for unstructured regions in degradation offers the following new insights: a) origin and identification of an intrinsic degradation signal in the substrate, b) identification of sequences in the native substrate that are likely to be responsible for direct interactions with the proteasome, and c) identification of critical rate limiting steps like exposure of the intrinsic degron and destabilization of an unfolding intermediate that are presumably catalyzed by the ATPases. Apomyoglobin emerges as a new model substrate to further explore the role of ATPases and protein structure in proteasomal degradation

## Introduction

Almost every cellular pathway involved in the biology of an eukaryotic organism is homeostatically regulated by the Ubiquitin Proteasome System (UPS) [1], [2], [3]. Impairment in the function of UPS components results in the accumulation of proteins leading to cellular stress and apoptosis [2]. Unlike other proteases, the proteasomes (26S) degrade fully folded proteins and generate short peptides and amino acids [4]. Under specific circumstances degradation is restricted to a single endoproteolytic cleavage to release intact functional domains [5], [6], [7]. Most proteins are normally tagged for degradation by a post-translational modification called ubiquitination while others do not require this modification [1]. Substrate recognition, binding/release, chain unfolding, translocation and degradation are common to both ubiquitin dependent, independent processes and to other ATP dependent compartmentalized proteases. Any of these steps can be rate limiting [8], [9], [10], [11].

Despite its long and well established role in cellular homoeostasis and recent clinical utility, many basic aspects of proteasomal degradation are largely unknown. Role of protein sequence, structure, thermodynamic and kinetic aspects of degradation is only beginning to be addressed. The complex architecture of the enzyme (26S proteasome) and the fact that not all proteins are amenable for degradation *in vitro* are major deterrents to such studies.

The major functional unit of the proteasome is the 26S holo complex made up of two modules- the 19S regulatory particles and the 20S proteolytic core [12]. The 20S proteolytic core is a central four ringed cylindrical barrel made up of seven membered α-β-α-β ring structure. Three types of catalytic sites, the trypsin-like (β2), caspase-like (β1) and the chymotrypsin-like (β5) are located within each β-ring. The outer α-rings are sandwiched by the 19S regulatory particles [1], [13]. The 19S regulatory particles are made up of at least 13 non-identical subunits, 6 of which are ATPases. Some of these subunits are responsible for substrate recognition via ubiquitin [14], [15]. At least one of the subunit is a deubiquitinating enzyme which releases the polyubiquitin chain before the substrate enters the proteolytic core. The ATPases are presumed to unfold and translocate the polypeptide chain into the 20S particles where proteolysis takes place. Access to 20S is restricted by a closed gate guarded by loops in the α-ring which restricts entry of even small peptides [16]. Assembly with the 19S regulatory particles opens the gate allowing access to the active site chamber formed by the β-rings. Even when the gate is open, diameter of the channel remains small, measuring about 13 Å which ensures that only unfolded proteins are committed for degradation [16].

Such a well-organized compartmentalized architecture of the proteasome as described above would indicate that purified intact 26S proteasomes must be self-sufficient to recognize and degrade any folded protein, ubiquitinated or otherwise. Surprisingly however, even ubiquitinated substrates are not amenable for degradation *in vitro* unless they also carry a degradation tag derived from long unstructured regions in other proteins. This is well established by pioneering studies from the Matouschek group using barnase and dihydrofolate reductase (DHFR) which are engineered to undergo ubiquitination and degradation through the N-end rule pathway [17], [18]. Barnase is a single polypeptide chain of 110 amino acids that forms three α-helices followed by a five-stranded anti-parallel β-sheet. DHFR is a protein made up of 187 residues that forms an eight stranded β-pleated sheet with four helices connecting the successive β-strands. Non-native long and short disordered regions derived from other proteins act as extrinsic degradation signals for these substrates. These and similar model systems have been useful in demonstrating the relative importance of ubiquitin and the degradation tag, the role of ATPases in local unraveling, and the effect of domain fusions on degradation [17], [18], [19], [20]. Another notable outcome from these studies is that proteasomes can degrade some proteins even when they are bound to ligands provided the degradation signal is directly followed by a surface helix or loop and not a beta strand.

Ubiquitin independent degradation has been clearly demonstrated for ornithine decarboxylase (ODC), which nevertheless, requires antizyme for recruitment to the proteasome. Although not ubiquitinated, DHFR could be artificially recruited to the proteasome by fusing an unstructured tag and a proteasomal subunit [10]. Again, in the absence of the unstructured region no degradation was observed. Inside the cells thymidylate synthase has been shown to be degraded in an ubiquitin independent manner with a half- life of about 12 h [21], [22]. Proteins with unfolded domains and large unstructured regions, and partially truncated proteins are substrates of 20S proteasome which are degraded in an ubiquitin independent manner [23].The few model systems used to study the mechanism of degradation of the eukaryotic proteasome has been catalogued in **Supplemental Table S1**.

In summary, to the best of our knowledge, all *in vitro* experiments so far have failed to establish the inherent ability of 26S proteasomes to recognize and degrade a folded protein in the absence of ubiquitin and/or extrinsic factors. Using apomyoglobin (apoMb), we provide first evidence for the natural ability of purified eukaryotic 26S proteasomes to directly recognize, unfold and degrade a globular protein in the absence of ubiquitin, extrinsic degradation tags or adaptor proteins. Using peptide panning studies and competition experiments we identify sequence elements within the substrate that are likely to be responsible for interaction with the proteasome. Using structure guided design, site directed mutagenesis, parallel biophysical studies and proteolytic susceptibility as a probe for protein dynamics we show that the mechanism of degradation followed by this small all helical protein is surprisingly reminiscent of ubiquitinated multi-domain proteins. We identify new rate limiting steps in degradation which involves substrate recognition, generation of intrinsic degradation signals and most likely melting of unfolding intermediates. The latter two steps are presumably catalyzed by the proteasomal ATPases.

## Results

### Choosing the model substrate and establishing its degradation by the eukaryotic proteasomes

We chose to test myoglobin as a model substrate because this protein is a small all helical protein that consists of 153 amino acids and exists both in a heme (ligand) bound holoform and a ligand free apo form. Presence of ligands or interacting partners may or may not protect proteins from degradation by the proteasomes [17], [18]. Crystal structure of holoprotein and NMR structure of the apoform are available [24], [25], [26]. Therefore, targeted protein manipulations and structural interpretations are possible. The thermodynamics and kinetic parameters of apoMb and the structure of the unfolding intermediates formed upon chemical denaturation have been fairly well characterized [27], [28], [29], [30], [31], [32]_ENREF_26. Information from such studies may provide insights into the mechanism of unfolding by the AAA ATPases of the 26S proteasome.

**Figure 1:**
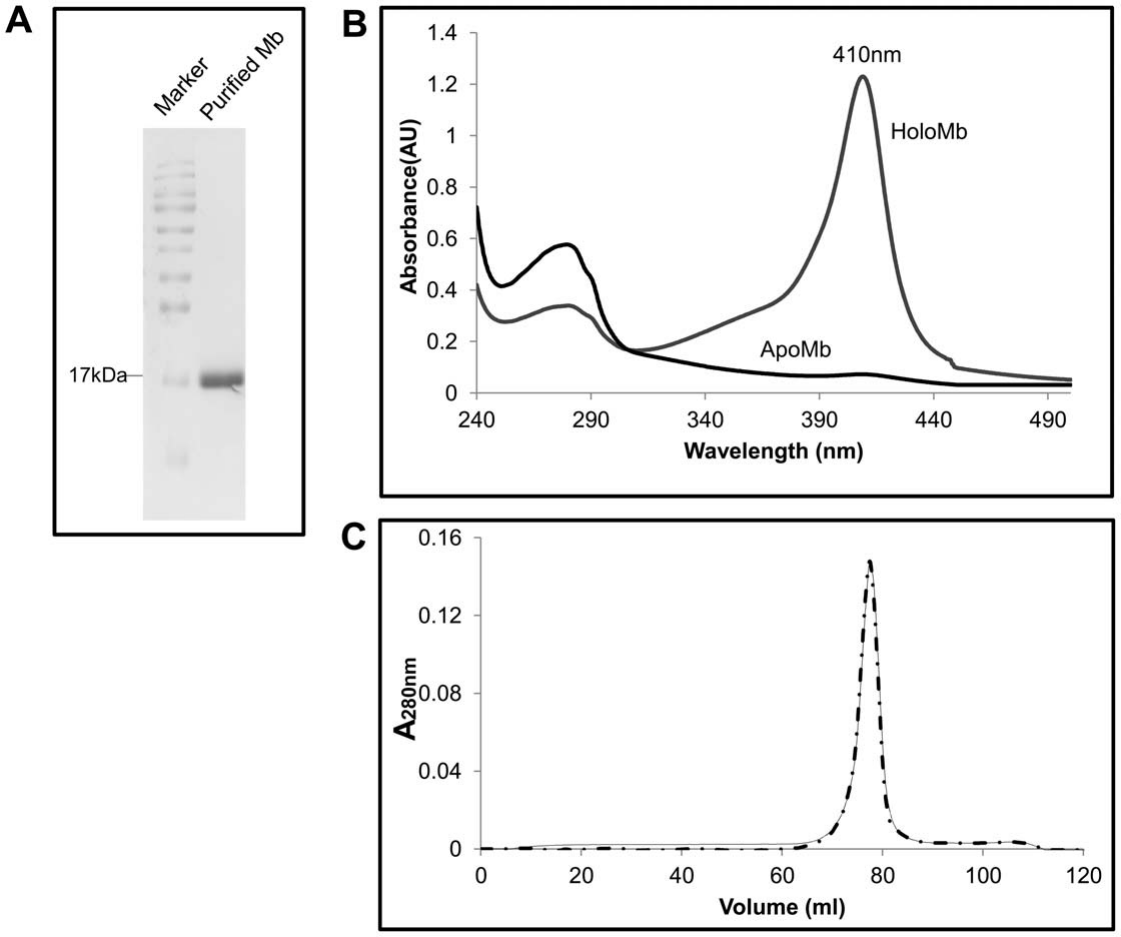
Purification and characterization of holo and apo myoglobin (Mb). (A) Mb was purified by cation exchange chromatography and was adjudged to be pure by SDS-PAGE analysis. (B) UV-visible spectrum of the holo and apoMb were recorded from 500 nm to 240 nm. A distinct Soret peak (410 nm) was observed in the holo form the intensity of which was reduced to about 95% in the apoMb indicating successful removal of heme. (C) Quaternary structure of apoMb was assessed by gel permeation chromatography. ApoMb (dash line) eluted at the same retention volume as the holo protein (solid line) demonstrating similarity in the protein fold.

Recombinant sperm whale myoglobin [33] was expressed in DH5α and purified by cation exchange chromatography. Protein was found to be pure by SDS-PAGE (**Figure 1A**). UV-visible spectrum of the protein showed the characteristic Soret absorption at 410 nm from the bound heme (**Figure 1B**). ApoMb was prepared from the holo protein as described elsewhere [34]. Heme removal was confirmed by loss of the Soret peak (**Figure 1B**). Upon size exclusion chromatography, apoMb eluted at the same retention volume as the holoprotein, indicating that ligand removal did not drastically affect the quaternary structure or the overall fold of the apoform (**Figure 1C**). Yeast 26S and 20S proteasomes were purified by affinity chromatography [35]. Assembly of the holo 26S and the 20S proteasomes were verified by native page and in-gel activity assay (**Supplementary Figure S1A**). Subunit composition was assessed by SDS-PAGE (**Supplementary Figure S1B**). Increase in fluorescence upon release of AMC from Suc-LLVY-AMC was monitored during purification of the proteasome.

**Figure 2:**
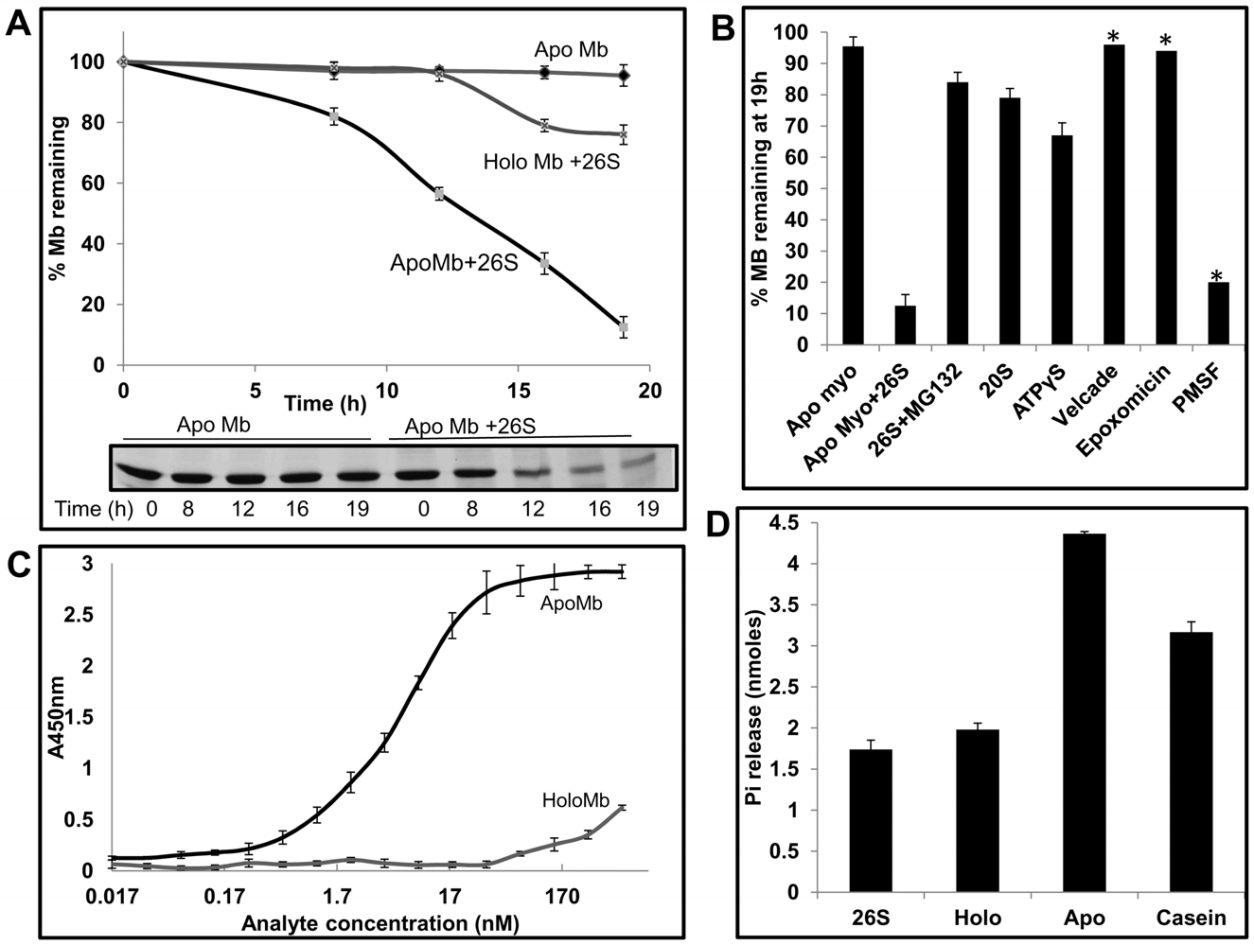
Purified yeast 26S proteasome recognizes and degrades apoMb *in vitro* in the absence of ubiquitin and any trans-acting element in an ATP dependent manner. (A) Purified 26S proteasomes were to able degrade apoMb but not the holoprotein. Proteins were incubated with 26S proteasomes at 37°C. Rate of degradation was followed by SDS-PAGE (inset) and quantified as described in methods. (B) Degradation of apoMb is dependent on the 19S regulatory particle and ATP. Purified 20S core particles were not able to degrade apoMb. Degradation by 26S proteasome was inhibited by MG132, epoxomicin and Velcade but not PMSF. No significant degradation was observed in the presence of ATPγS, the non-hydrolysable analog of ATP. (C) ApoMb and not the holo form is recognized by the 26S proteasomes. ApoMb and holoMb were incubated with immobilized proteasome and detected using anti-Mb antibody. (D) ApoMb and not the holo form is able to stimulate the ATPase activity of the 26S proteasomes. Data represent the mean values of at least three independent experiments ±S.D. * Single experiment.

Using these purified components, ability of the 26S proteasome to degrade apoMb was tested. Degradation was monitored by the disappearance of band intensity of apoMb on a 15% SDS-PAGE. Given that the protein is all helical, we expected it to be degraded rapidly by the proteasome. However, degradation is a slow process and it takes ∼12 h for the proteasome to degrade 50% of the substrate (**Figure 2A**). Degradation was inhibited by MG132, an active site inhibitor of the proteasome (**Figure 2B**). A more specific inhibitor of the proteasome like Velcade and an irreversible inhibitor of the proteasome, epoxomicin, also inhibited degradation (**Figure 2B**). PMSF, a serine protease inhibitor even at 1 mM did not affect degradation to any measurable extent (**Figure 2B**).

### Degradation of apoMb is catalyzed by intact 26S proteasomes by direct recognition of the substrate

In order to check if the ATPases were engaged in the degradation process, we tested the energy requirement for degradation. There was negligible degradation in the presence of ATPγS (**Figure 2B**). Energy from ATP hydrolysis is presumably required for chain unfolding and translocation. To test whether degradation was actually mediated by intact 26S proteasomes and to ensure that they remained stable, proteasomes incubated in the assay buffer (with 3 mM ATP and 3.5% glycerol) at 37°C for 12 h, were analyzed by native page electrophoresis. In-gel activity assay as well as coommassie staining was performed. The holo complex was essentially intact and 20S proteasomes were undetectable. A very small signal corresponding to singly capped 26S proteasomes was detectable in the in-gel activity assay (**Supplemental Figure S2A**). Moreover, proteasomes thus incubated retained ∼88% activity as measured by the release of Amc from Suc-LLVY-AMC (**Supplemental Table S2**). Isolated 20S proteasome was unable to degrade apoMb emphasizing the importance of 19S regulatory particles in the pre degradation processes (**Figure 2B**).

While our assumption that the ligand bound holoprotein would be more resistant to degradation proved correct, the reason for its stability was not quite obvious. A pertinent question to ask is whether the proteasome recognizes the holoprotein at all. We standardized an Enzyme-linked immunosorbent assay (ELISA) to probe the interaction between substrate and the proteasome. ApoMb was found to bind with high affinity to the immobilized proteasome (Kd = 3.5±1 nM), while the holoprotein did not bind to any appreciable extent (**Figure 2C**). In contrast to the 26S proteasomes, the 20S proteasomes seemed to bind to both holo and apoMb, but binding was not saturated (highest concentration tested was 580 nM) (**Supplemental Figure S3**). As mentioned above, 26S proteasomes remain intact over prolonged incubation and dissociation into 20S if any is undetectable. Even if present they are unlikely to compete for binding due to huge affinity differences between the 20S and 26S proteasomes.

A well-known property of substrates recognized by chaperones and AAA ATPases [36], [37] of the proteasome, is their ability to stimulate the ATPase activity. While apoMb was able to stimulate the ATPase activity by two fold (**Figure 2D**), the holoprotein commensurate with its failure to be recognized by the proteasome, had no effect on the ATPase activity. Taken together these results indicate that the degradation we observe is due to intact 26S particles that directly recognize the apo form of Mb. The ATPases must catalyze unfolding of the protein.

Stimulation of ATPase activity by apoMb was at a much faster time scale (in the order of minutes) and during this time, the protein was completely stable. These results indicate that despite high affinity binding, and eliciting a response from the proteasome, a substrate can be released prematurely. It seems that binding and down-stream events like chain unfolding and translocation must be coupled for the encounters to be productive. Any of these steps could be rate limiting.

### Structural elements involved in degradation

To determine the structural basis of degradation of apoMb, we compared the available crystal structure of the holo protein (PDB ID 2JHO) and the NMR structure of the apoform [25]. Removal of heme induces prominent conformational changes in apoMb. Most dramatic changes are seen within the EF-loop, the F-helix, the FG-loop, and the first few residues of the G-helix. This region (Lys 78-Phe 106-initiator Met is excluded in numbering) located almost in the middle of the protein exists in a conformational equilibrium between a well folded α-helical structure and a disordered ‘loop’ (unassigned resonances) [25]. This transition from helix to disordered state would be reflected in the CD spectra [34].

**Figure 3:**
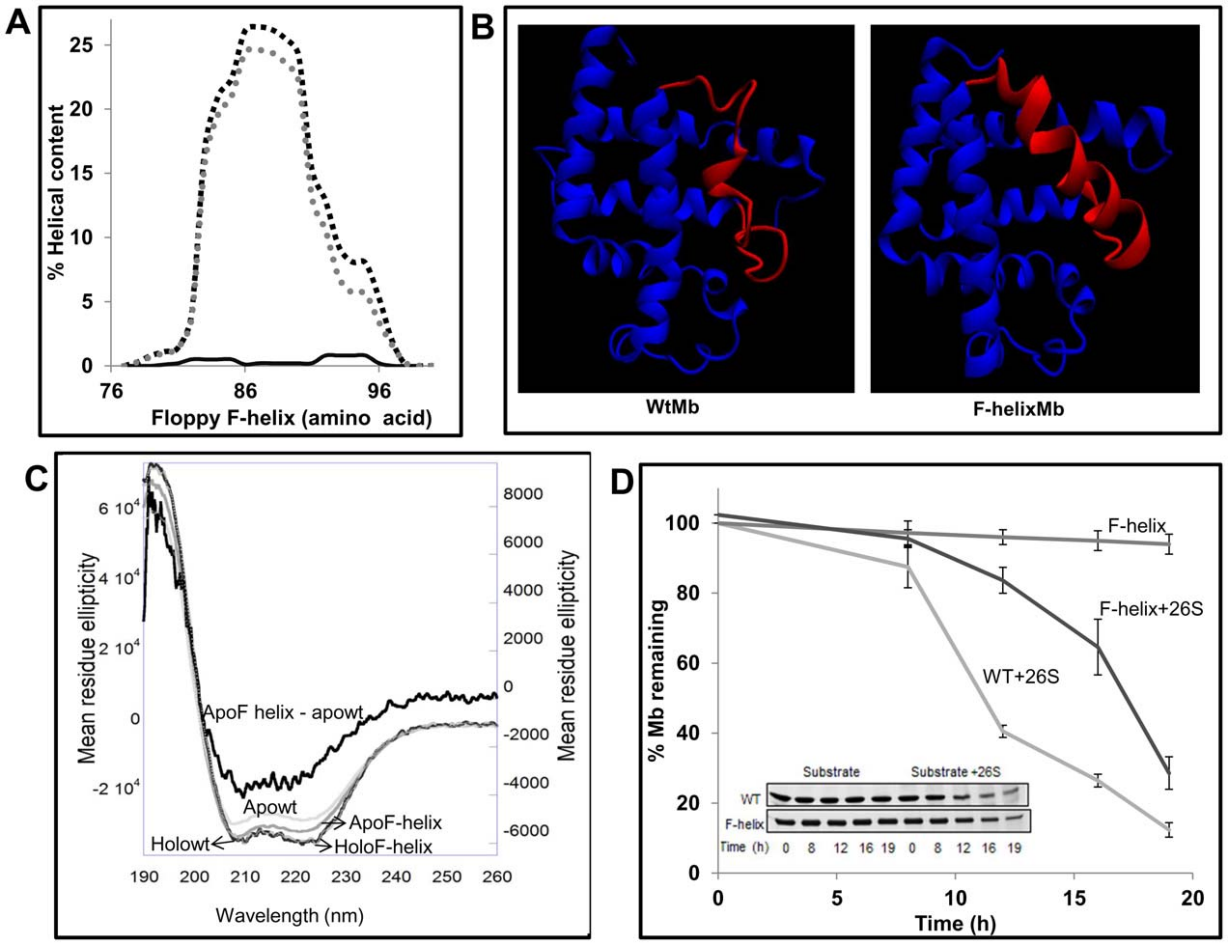
Floppy F-helix is crucial for the degradation of ApoMb. (A) AGADIR prediction (parameter, pH 7.5, Temperature 273K, Ionic strength 0.15 M) of the helical propensity of floppy F-helix. Wt sequence (solid line), G80AP88AS92AH97E (dashed grey) and G80AP88AS92AH97N (dashed black). (B) MD simulation of wt apoMb and the F-helix mutant. The wt sequence melts immediately at 400 K while the F-helix mutant remains stable even at the end of 2.8 ns simulation. (C) To verify the role of floppy F-helix exposed upon removal of heme, helix stabilizing mutations were introduced. Far-UV CD spectrum shows that the Apo F-helix mutant has enhanced secondary structure as compared to the wt ApoMb. The difference spectra were obtained by subtracting the spectra of apowt from apoF-helix (MRE values are on Y2). (D) Apo wt and apo F-helix proteins were incubated with proteasome. The rate of degradation was followed by SDS-PAGE (inset) and quantified as described in methods. Data represent the mean values ±S.D of at least three independent experiments for wt apoMb and five independent experiments for F-helix mutant. Remarkably, stabilization of F-helix rendered ApoMb more resistant to degradation by the proteasome.

We compared the secondary structure of holo and apoMb by recording the CD spectra in the far UV region (**Figure 3C**). The Mean Residue Ellipticity (MRE) at 222 nm (a reliable measure of helical content) [38] of the holo protein is −33053.7 deg cm^2^ dmol^−1^ and that of the apowt is −26943.8 deg cm^2^ dmol^−1^. There is 18% reduction in the helical content in going from the holo to the apo form. A 20% difference between the two has been reported by previous investigators [34].

Myoglobin has eight helices of varying length. As per the crystal structure, out of 153 residues, 128 lie in the helical region. Based on the linear relationship between CD and chain length, fractional contribution of each helix to the total helical content can be estimated. If the effects of conformational changes or mutations are known by other methods like crystal structure or NMR, one may attribute the differences in CD to specific structural changes. As noted before a floppy F-helix in myoglobin is exposed upon heme removal. Although the F-helix *per se* spans only from 82 to 96 residues (15 residues), for simplicity, the entire 78–106 disordered region will be referred to as the floppy F-helix. With this definition, 22 residues from the floppy F-helix would contribute to about 17% (22/128*100) of the total helical content of Mb. Since the experimentally observed difference in the helical content between holo and apo forms is ∼18%, 94% of this loss may be attributed to the helix to disorder transition in the floppy F-helix.

Based on the reported mandatory role of unstructured regions in the initiation of degradation, we hypothesized that floppy F-helix must be a key determinant in the degradation of apoMb. The floppy helix may either act as a recognition element and/or as an initiator of degradation. If so, stabilizing the helical structure in this region may alter the half-life of apoMb. To do so, mutations were designed with the intent to induce helicity and ensure that once formed the helix remains stable in the absence of heme. G80 in the EF-loop, Pro 88 and Ser 92 within the helix were replaced by Ala. The FG-loop is formed by KHKI (96–99) residues. Because of the presence of a positively charged cluster within this loop, a glutamic acid residue in the place of His97 would help in diffusing the repulsive forces and help in stabilizing the helical structure. When the sequence with the G80A/P88A/S92A/H97E replacement was analyzed using AGADIR [39], [40], [41], a program that provides residue level helical content (based on helix/coil transition theory) there was a clear increase in the helicity between 78–106 residues. When His 97 was replaced by Asn (G80A/P88A/S92A/H97N), there was a fractional increase in the helical content compared to the Glu mutant (**Figure 3A**). We therefore chose to test the G80A/P88A/S92A/H97N mutant for degradation by the proteasomes. Quick molecular dynamic simulations of the proteins at 400 K for 2.8 ns (**Supplemental method S1**) showed that the designed F-helix is indeed more stable as compared to the wild type sequence which melts almost immediately (**Figure 3B**).

The secondary structure of the holo and the apo forms of F-helix mutant were compared with the wt apoMb. There was no detectable difference between the two holo forms. As compared to apowt, the apo F-helix mutant showed substantial enhancement in the α-helical content (9±2%) (**Figure 3C**). As described before experimentally observed difference in helicity of about 18% between wt holo and apoMb could be attributed to the loss in structure of the floppy F-helix. Therefore the designed mutations by primarily stabilizing the helical conformation in the floppy F-helix seem to bring the structure of the apo protein to 50% of the holo form. With a helical content that lies in between that of holoMb and apowt, the F-helix mutant seems to be an intermediate in the folding/unfolding pathway.

Stability of the protein was measured by following the secondary structural changes as a function of temperature. The temperature at which 50% of the protein is present in the unfolded form (Tm) was calculated. Tm of wt protein and the mutant were almost identical (65°C) (**Supplementary Figure S4A**). In order to check if the mutations had an effect on the tertiary structure of the protein, tryptophan fluorescence was measured. The emission maximum and the fluorescence intensity were almost identical in both the wt and mutant proteins (**Supplementary Figure S4B**). The results indicate that the overall fold of the protein is not affected by mutation.

To further verify that the mutations had a major effect on the secondary structure rather than on the global stability of the protein, we compared the thermodynamic stability of wt apoMb and the F-helix mutant by subjecting them to urea denaturation at pH7.5 (pH at which degradation is performed). At near neutral pH (6–7) apoMb undergoes two state transitions [34], [42], [43]. We observed the same and using the equation ΔG = ΔG(H_2_O)-m[urea] (ΔG(H_2_O) is ΔG in the absence of urea and m, the dependence of ΔG on urea), the free energy of stabilization was calculated [44]. It is clear that the ΔG and the urea concentration required to unfold 50% of the protein are very similar. Thus mutations in the F-helix region seem to primarily stabilize the secondary structure in the floppy F-helix without affecting the overall stability of the protein (**Table 1**).

**Table 1:** Thermodynamic parameters from urea denaturation.

	Wt	F-helix
ΔG (H2O)(kcal mol^−1^)	6.14±0.25	6.12±0.25
***m***(kcal mol^−1^M^−1^)	−1.41±0.05	−1.49±0.06
C_m_ (M)	4.3	4.09

The urea-induced unfolding was analyzed in 20 mM po4 buffer pH7.5, assuming ΔG has a linear dependence on the urea concentration: ΔG = ΔG(H_2_O)-m[urea], where ΔG(H_2_O) is an estimate of the value of ΔG in the absence of urea and m is a measure of the dependence of ΔG on the urea concentration. The values for each data point are averages calculated on the basis of at least four independent experiments. Errors shown are derived from the curve-fitting calculations.

We checked the effect of these mutations on degradation of apoMb and found that as predicted, stabilization of the F-helix altered the half-life of apoMb. It takes 16 h for the proteasome to degrade 50% of the F-helix mutant (**Figure 3D**) while the same is achieved in 12 h in the case of the wt protein. This result reflects the fact that the proteasome is sensitive to local secondary structural alterations in this ubiquitin independent degradation of single domain all helical protein.

To further prove that the mutations indeed stabilize the floppy F-helix we performed limited proteolytic digests, a technique used as an index of protein conformational status and dynamics [45]. Accessibility of a putative cleavage site is an important determinant in endoproteolysis and is a parameter that can be quantified. Recently we had combined this quantifiable parameter called Solvent Accessible Surface Area from crystal structure of proteins and sequence specificity of proteases to developed an algorithm called PNSAS (Prediction of Natural Substrates from Artificial Substrate of Proteases) to predict natural substrates of endoproteases [46].

**Figure 4:**
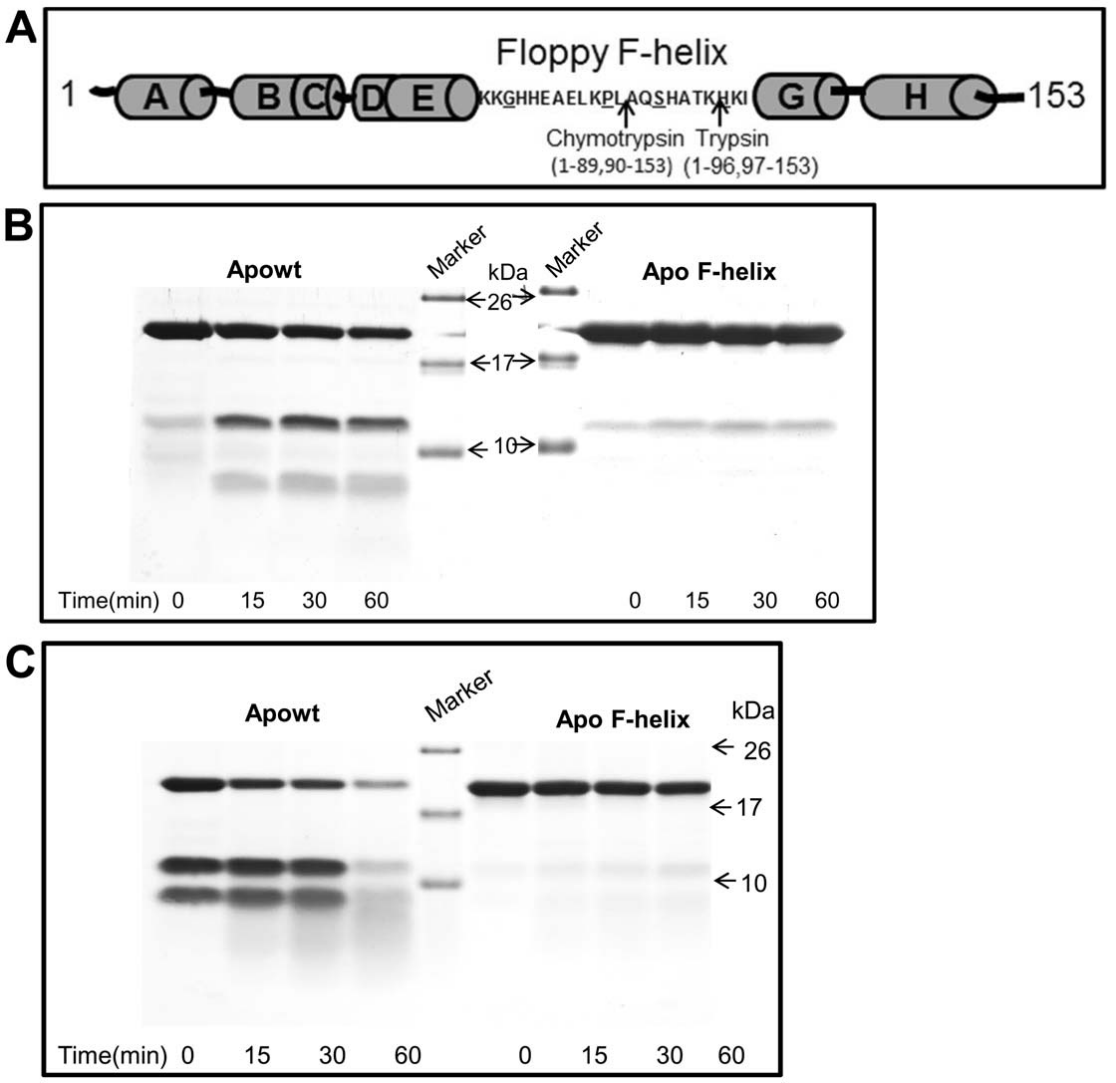
Limited proteolysis demonstrates the presence of an unstable and stable F-helix in wt and F-helix mutant respectively. (A) Cleavage sites of trypsin and chymotrypsin on Mb are diagrammatically represented (in F-helix underlined amino acids are mutated). Trypsin (B) and chymotrypsin (C) were added to wt and F-helix mutant. Aliquots at various time intervals were analyzed by Tricine-SDS Page. Wt protein is cleaved by chymotrypsin as soon as the enzyme is added (0 min). These fragments are not contaminant in the preparation as can be seen from the purity of Mb in Figure 1. Substrate alone controls were stable (data not shown).

Initial nicking of apoMb by trypsin or chymotrypsin results in the generation of two fragments through a cut within the floppy F-helix. Trypsin cuts between Lys 96 and His 97 while chymotrypsin nicks between residues Leu 89 and Ala 90 [47] (**Figure 4A**). Therefore, if mutations have indeed stabilized the floppy F-helix, the F-helix mutant should be relatively resistant to cleavage by these enzymes. Addition of trypsin or chymotrypsin to wt apoMb generates two fragments. As compared to trypsin (**Figure 4B**), chymotrypsin (**Figure 4C**) cleaves wtapoMb at much faster rate and the cleaved products can be seen immediately after addition of chymotrypsin. The cleaved products were analyzed by MS and they correspond to the expected fragment size (data not shown). With time further hydrolysis of the fragments takes place. In sharp contrast, the F-helix mutant is highly stable and very little of the cleaved products were seen upon prolonged incubation (**Figure 4 B and C**).

To test whether mutations in the floppy F-helix had an effect on the affinity of the protein for the proteasome which may explain the increase in half-life, an ELISA was performed. Binding of F-helix mutant to the proteasome was not adversely affected (Kd = 0.57±1 nM *vs* 3.5±1 nM for the wt) (**Table 2**) indicating that the residues mutated are not directly involved in interaction. Taken together these results provide direct proof that the disordered F-helix is stabilized by mutation and therefore conformational changes involving this helix must be responsible for the effect on degradation.

**Table 2:** Effect of apoMb secondary structure, Trp environment, melting temperature (Tm) and affinity (Kd) on proteasomal degradation.

Protein	% Helix*		Relative Trp fluorescence	Tm (°C)	Affinity to proteasome(Kd nM)#	Half-life (h)
	By 222 nm$	SELCON3©				
Holowt	92	94	ND	80	ND	ND
ApoWT	76	79	1	65	3.5±1	12
V10CA-helix	74	79	0.82No shift	60	1.25±0.3	12
T39CC-helix	74	79	0.821 nm red shift	63	19.5±7	12
StabilizedF-helix	85	87	1No shift	65	0.57±0.1	16
L104CG-helix	60	62	0.821 nm blue shift	56	0.6±0.5	6–7
L115CG-helix	58	58	0.831 nm blue shift	56	0.63±0.3	7–8
M131CH-helix	75	79	0.892 nm red shift	55	45±20	12

ND = not determined.

* 2-4% variation in helicity was observed in three independent experiments.

© Data for CONTIN (not shown) was similar to SELCON 3.

# Kd represents mean value of three independent experiments ±S.D.

$ Fractional helical content = ([θ] 222–3,000)/(−36,000–3,000) [38].

### Recognition element in apoMb

The floppy F-helix may either serve as a recognition element or as an initiator of degradation. To test whether this region is involved in interaction with the proteasome and to map other proteasome binding sites on apoMb, 15 amino acid sequences with 8 amino acid overlap between two contiguous regions were selected. These peptides were synthesized with biotin at the N-terminus and screened for binding to the proteasome (**Supplementary Figure S5A**). Peptides with the sequence from A-helix bound strongly to the proteasome. Two other peptides which encompassed B-helix and the CD-loop showed weak affinity.

**Figure 5:**
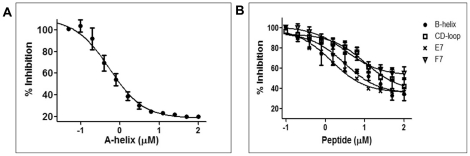
Floppy F-helix sensitizes the proteasome for the presence of substrate and the interaction is reinforced by N-terminal helices. Overlapping peptides panning the entire length of myoglobin were tested for binding to the immobilized proteasome by ELISA. (A) A-helix peptide which bound strongly to the proteasome was tested for its ability to compete with apoMb. This peptide brought about 80% inhibition at 3 µM concentration. (B) The B-helix, CD-loop, E7 and F7 peptides which share sequences with the floppy F-helix also inhibited the binding of apoMb. However they were considerably less potent than the A-helix peptide. All incubations were done in the presence of 100 nM MG132. Data represent mean values of at least three independent ±S.D. For E7 S.D. is not plotted for clarity.

To rule out the possibility that the lack of binding of some of the other peptides could be due to their degradation by proteasome, we used 100 nM of MG132 during all incubation steps in ELISA. Interaction of peptides derived from A-helix, B-helix and CD-loop region were not significantly altered but peptides E7 (69–83, forming part of the C-termini of the E-helix and the EF-loop) and F7 (90–104, forming C-termini of the F-helix, FG-loop and N-termini of the G-helix) showed measurable binding. These peptides were then tested in a competition assay. Commensurate with the direct binding studies, the A-helix peptide inhibited binding of apoMb with a Ki of 0.8±0.4 µM (**Figure 5A**). At 100 µM concentration, B-helix and the CD-loop peptides brought about 40 and 48% inhibition in the binding of apoMb to the proteasome respectively (**Figure 5B**). At the same concentration, E7 and F7 brought about 34 and 65% inhibition respectively (**Figure 5B**). In sharp contrast, the A-helix peptide singularly achieved near complete inhibition with 80% of interaction between apoMb and the proteasome abrogated at a concentration of ∼3 µM. Thus bulk of the binding energy between apoMb and proteasome seems to be derived from the more structured A-helix region. Using a binding assay that is independent of proteolysis and ATPase stimulation, we have been able to map the possible interactions between different regions of apoMb and the proteasome.

A specifically designed 23 residue peptide covering the sequence from 77–100 in the F-helix region could not inhibit binding of apoMb to the proteasome at the concentrations tested (data not shown). Barring a short stretch of residues between 69–77 that forms the C-termini of E-helix, the sequence of floppy F-helix (78–106) overlaps with the E7 and F7 peptides. Therefore apoMb is likely to interact with the proteasome *via* weak interactions originating from the residues at the C-termini of the E and the N-termini of the G-helices. This region is highly disordered. It is likely that this floppy F-helix enters the central channel of the proteasome in the form of a loop and acts as an initiator of degradation.

### Degradation is accelerated when mutations made in adjacent helices destabilize the secondary structure

The 29 residue F-helix loop in apoMb is not long enough to enter the active site chamber. Considering each residue length to be ∼3.8 Å, a 29 residue polypeptide when stretched extends to about 11 nm. In the form of a loop the length of this region would be reduced to about 5.3 nm. The shortest distance from the surface of ATPases in the 19S, to the first active site in the β-ring is about 15 nm long. These structural constraints necessitates that the neighboring helices should unwind to create an extended loop or termini consisting of at least 58 more residues so that the polypeptide can reach the sequestered active site to initiate proteolysis. It is quite likely this process is a rate limiting step in the degradation of apoMb.

**Figure 6:**
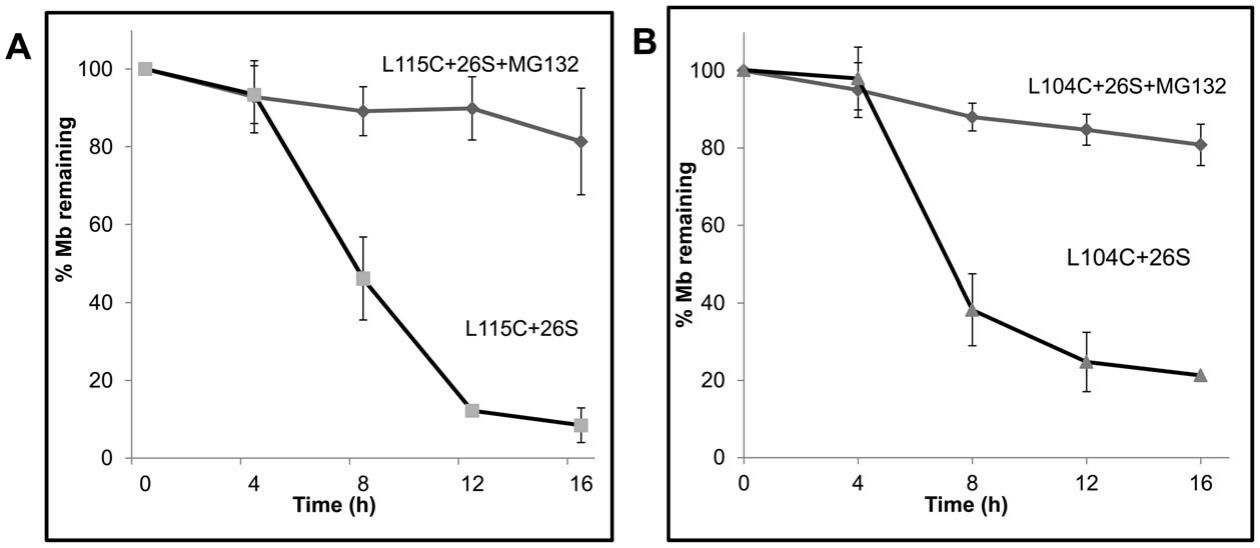
Mutation of buried Leu residues in the G-helix shortens the half-life of apoMb. (A) Apo L115C and (B) apo L104C mutant proteins were incubated with proteasome in the presence or absence of MG132. Rate of degradation was followed by SDS-PAGE and quantified as described in methods. Data represent mean values of at least three independent experiments ±S.D.

Mutations that can propagate disorder in any of the adjacent helices are likely to enhance the length of the loop. We mutated one buried residue within each helix with the hope that they may alter the local secondary structure. We found that some of these mutants had a clear effect on the helical content of apoMb but minimal effect on the tertiary structure (**Table 2**). These mutant proteins were V10C (A-helix), T39C (C-helix), L104C (G-helix), L115C (G-helix) and M131C (H-helix). There was ∼20% reduction in the intensity of Trp fluorescence in all the Cys mutants. The Tm of V10C and T39C mutants were comparable to that of the wt. Their helical content and half-life were also similar to that of the wt protein. On the other hand, the Tm of L104C, L115C and M131C were reduced by 10°C. However, only the L115C and the L104C mutations had an adverse effect on the helical content (less by 21 and 17% respectively) (**Table 2**). Notably, these mutations had a profound effect on the rate of degradation and the half-life of the proteins was estimated to be 7.5 h and 6.5 h respectively (**Figure 6** **A and B**). This amounts to half the time required to degrade 50% of the wild type protein. The M131 mutant with lower Tm but unaltered secondary structure was degraded at similar rates like the wt protein.

Careful look at the crystal structure shows that L104 is present in the N-terminal region of G-helix, but forms part of the disordered region (78–106) in apoMb. In the holo protein, L104 interacts with residues in the H-helix. L115C is in the C-terminal half of the G-helix and interacts with residues in the A-helix. Compared to apowt, the respective loss in the helical content of L104C and L115C mutants is 17 and 21%. Likewise, the respective loss in helical content as compared to holo protein is 32 and 36%. As noted before, the difference between the holoMb and wtapoMb is 18% which is largely accounted for by the loss in the helical content of the floppy F-helix. Therefore, additional losses in these two Leu mutants must involve other regions of the protein. This is reflected in the limited proteolysis experiments which show that the sites for cleavage are more readily accessible in both the Leu mutants as compared to wt apoMb (**Supplemental Figure S6A**). The ‘disordered conformation’ of the F-helix is stabilized in the mutant proteins rendering it more susceptible to proteolysis. While disruption of helix-helix packing and co-operative nature of folding could also account for the loss in helical content, the primary driving force is likely to involve propagation of the disorder into adjacent helices which facilitates degradation.

## Discussion

In order to investigate the sequence and structural requirements for the degradation of an ubiquitin independent substrate by the eukaryotic proteasomes, and identify the rate limiting steps, we developed an *in vitro* model system composed of affinity purified yeast 26S proteasomes and pure apoMb.

### Mechanism of degradation of apoMb and the structural determinants involved

**Figure 7:**
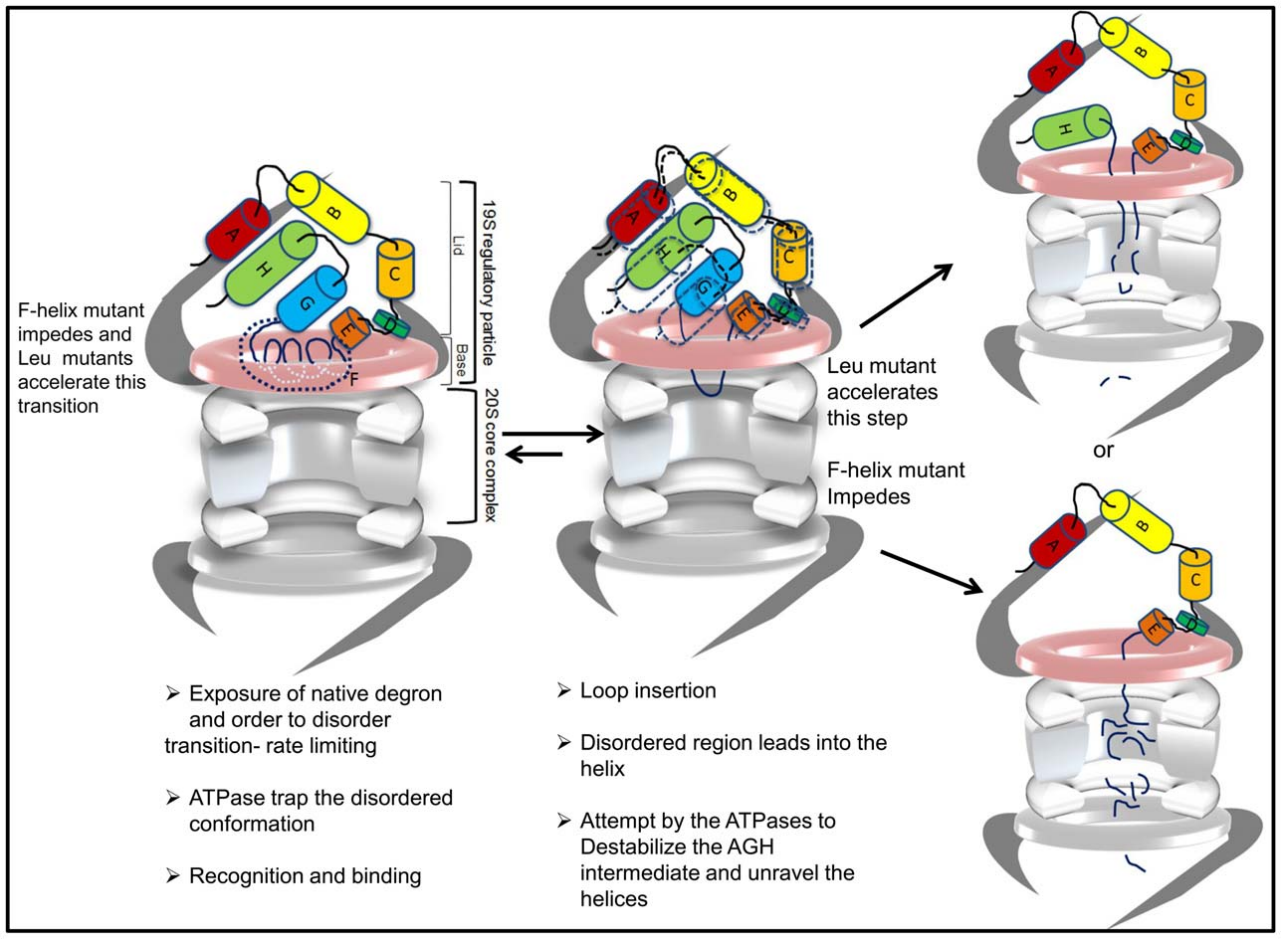
A model for the mechanistic steps involved in the degradation of apoMb based on structural changes in mutations used as snap shots. Removal of heme exposes a previously buried F-helix which is in a dynamic equilibrium between a partially folded and unfolded structure. This transition is a rate limiting step. Exposure of this floppy helix sensitizes the proteasome to the presence of the substrate. ApoMb is anchored to the proteasome by interactions primarily through the A-helix. Additional interactions stabilize the enzyme-substrate complex. Degradation is primed by the insertion of the floppy helix in the form of a loop into the central channel that runs across the proteasome. An intermediate composed of AGH helices is likely to be formed. Melting of this intermediate by the ATPases to generate an unstructured region long enough to reach the active site is a likely rate limiting step. Mutations can stabilize or destabilize this unfolding intermediate affecting the rate of degradation.

The unraveling mechanism of unfolding would predict that if the degradation signals in proteins lead directly into helices or surface loops then they are likely to be easily degraded. In such easily digested proteins this is found to be true even when the substrate is bound by a ligand [17], [18]. ApoMb is a ligand free small single domain protein. The floppy F-helix which acts as the intrinsic degradation signal is flanked by helices and loops. Nevertheless degradation is surprisingly a slow process. We believe that there are several reasons for this unexpected behavior and enumerate them below. The structural changes in the mutant proteins and their effect on degradation are used as snap shots to reconstruct the key events in degradation of apoMb (**Figure 7**).

### Destabilization of the unfolding intermediate

Slow degradation of apoMb raises the possibility that under these experimental conditions there is a slow conversion of conformationally unstable apoMb to non-native/unfolded forms which may be the actual substrates. While this is a debatable issue, we believe it is unlikely for the following reasons. 1. Free energy of stabilization of the wt and the F-helix mutant are more or less the same (**Table 1**) and yet the F-helix mutant is degraded slowly. 2. Despite a low Tm, mutant with unaltered secondary structure (M131C) has the same half-life as the wild type protein. 3. Secondary structure and the fluorescence properties of apoMb are not dramatically altered even after 12 h of incubation (**Supplemental Figure S2B**). Even more importantly, if apoMb was unstable and the unfolded forms were to accumulate, this would be more pronounced in L115C or L104C mutants which in their native state are less structured than the wt protein. We did not detect any structural changes in these proteins with time (**Supplemental Table S3**). In addition one would presume that 20S proteasomes which can act on unstructured proteins must be able to degrade the accumulating unfolded forms. We tested the degradation of L104C mutant and β-casein (an unstructured protein) by purified 20S proteasomes. While most of the casein is degraded by 3 h, L104C is stable even after 8 h of incubation with 20S proteasome, a time point when more than 50% of L104C is degraded by the 26S proteasome (**Supplemental Figure S6B**).

The only likely explanation that would account for the observed slow degradation of apoMb is the formation of an unfolding intermediate that is resistant to degradation. Reasons are the following.

Limited proteolysis experiment suggests that the floppy F-helix region in apoMb is readily accessible to trypsin or chymotrypsin. Due to the compartmentalized nature of the proteasome, mere exposure or accessibility of the F-helix region alone is not sufficient for proteolysis. A disordered region that is long enough to reach the sequestered active site located deep inside the catalytic chamber is necessary for initiating proteolysis. Creating such a long segment (presumably catalyzed by the ATPases) would constitute a key rate limiting step. It is well known that the denaturant induced unfolding of apoMb proceeds through a stable long lived intermediate formed by AGH helices [48], [49], [50]. Since the mechanism of unfolding of apoMb by the proteasome seems to follow a process termed chain unraveling rather than global unfolding, it is possible that a similar intermediate is populated during this process. Destabilizing such an intermediate would be a key rate limiting step and may explain the unexpected slow rate of degradation of even the wt apoMb. Mutant proteins like L104C and L115C with a more destabilized structure are degraded relatively faster because the unfolding intermediate must be less stable in these mutants. It is likely that such an intermediate would be more stable in the F-helix mutant that could account partly for the increased half-life.

### Creation of the disordered F-helix

The slow degradation of wt apoMb is altered upon stabilization of the F-helix or when more unstructured regions are created in adjacent helices by mutation. Since the floppy F-helix oscillates between a folded and floppy conformation, equilibrium would favor the more stable form. Since a denatured or disordered conformation seems necessary for proteolysis, this conformational distribution favoring the folded state of F-helix may explain the delay associated with degradation of the mutant.

Recently the NMR structure of F-helix stabilized apoMb has been solved. Two mutants that could enhance the secondary structure in the F-helix were designed using AGADIR. One of the mutant protein F2 (P88K/S92K double mutant) has the same free energy of stabilization like the wt protein [43]. The predicted enhancement in secondary structure of F2 was confirmed by CD and NMR resonances that were undetectable in apowt could now be assigned to the F-helix region. These NMR structures provide support for our claim that mutations in F-helix (G80A/P88A/S92A/H93N) which results in enhanced secondary structure, (AGADIR, CD and limited proteolysis) with no apparent effect of global stability (similar ΔG and Tm like wtapoMb), is most likely due to the conversion of the disordered floppy F-helix to a more stable form. As indicated by the limited proteolysis experiment melting of this F-helix in the mutant to a disordered structure is a slow process. It is likely that conversion of the F-helix to a disordered state and therefore creation of degron are catalyzed by the ATPases of the proteasome. This may explain some of the delay observed in the half-life of the mutant as compared to the wt protein. Notably no detectable degradation takes place when the helix is buried in the holoform. Thus, formation of an endogenous degron seems to be a key rate limiting step.

### Other Rate limiting steps in degradation

#### Recognition is one of the key rate limiting steps

A direct interaction between the proteasome and its substrate as well as the determinants of protein-protein interactions between the two have not been reported till date. Using overlapping peptides that pan the entire sequence of apoMb, sequences that can directly interact with the proteasome and/or compete with apoMb for binding were identified. Results from these experiments identify the probable regions within the substrate that are important for interaction with the proteasome. Peptides derived from the F-helix region are weak inhibitors of interaction. One of the main reasons for the failure of the proteasome to degrade holoMb is the very weak affinity of interaction primarily due to unavailability of the floppy F-helix. Taking these two observations together we believe that initial encounters between apoMb and the proteasome is most likely mediated through the floppy F-helix which sensitizes the proteasome and primes the substrate for degradation. This is further supported by the fact the mutant despite having a more stable F-helix than the wt apoMb, is able to interact with the proteasome. Furthermore, since sequence from A-helix is not only able to directly bind to the proteasome with high affinity, but is also able to completely neutralize the binding of apoMb, the bulk of the interaction between apoMb and proteasome is likely to be mediated by the A-helix aided by sequences from B helix and CD loop.

Available literature suggests that the conformation and the stability of the A-helix are very similar in the holo and apoprotein [25]. The structure of the A-helix region seems unaltered between the holo and the apoform and yet holoMb is not recognized to any significant extent by intact 26S proteasome. This seemingly contradictory observation can be reconciled if one imagines that the primary interaction is mediated by the disordered F-helix exposed only in apoMb (EF loop and FG loop as seen by the peptide panning studies). This probably alters the conformation of A-helix in a manner that allows binding to the proteasome. The bound conformation of the A helix must be different from that of unbound apoMb. A peptide which is free and flexible may adopt the bound conformation that would explain its ability to inhibit apoMb. Recognition through F-helix region seems mandatory for other interactions to take place.

### How does the rate of degradation of different mutant proteins correlate with their binding affinity?

One expects a direct correlation between affinity and rate of degradation. The maximum difference in binding affinity between apowt and any of the mutant protein studied is within an order of magnitude and the correlations are direct in some cases and inverse in others. M131C and T39C with ∼7–10 fold lower affinity are degraded to the same extent as the wt (**Table 2**). They have similar helical content like the wt apoMb. The F-helix mutant with ∼6 times more affinity than the wt is degraded slowly. This is likely due to a) the slow transition between the folded and unfolded forms of the floppy F-helix and b) the presence of a stable unfolding intermediate. The two Leu mutants with 6 fold higher affinity are degraded much faster than the wt protein. Thus destabilization of the adjacent helices and/or the unfolding intermediate seem to be the driving force that accelerates degradation rather than affinity per se.

Analysis of the crystal structure of the holo protein indicates that T39 in C-helix interacts with residues within the CD loop. M131 interacts with two residues in the A-helix and one in the G-helix. Since our competition experiments with the peptides indicated that the A helix and CD loop are involved in interaction with the proteasome, it seems that T39 and M131 may have a role to play in influencing this interaction. Based on the extensive interaction between the helices of apoprotein and the proteasome uncovered by our peptide panning and competition experiments, cooperative nature of these interactions cannot be ruled out. Moreover existence of additional interactions that are not revealed by peptide panning experiments cannot be ruled out.

Taken together these results indicate that the major influence on the rate of degradation seems to be the folded state of the helices and the proteasome is tolerant to small changes in binding affinity. But exposure of the F-helix or the intrinsic degron which is critical for initial recognition constitutes the first major rate limiting step in degradation. Mutations of the residues in the A-helix and identification of the subunit/s of the 19S regulatory particles involved in recognition of apoMb would further enhance our understanding of the mechanism involved in degradation.

### What are the general aspects of ApoMb degradation?

ApoMb is likely to represent ligand free form of proteins and proteins which have dissociated from their binding partners or subunits. In such cases previously buried interacting region may become exposed. Many proteins contain disordered loops within their structure that are highly flexible. Some if not all may be responsible for degradation by the proteasome. L104C and L115C mutants may represent molten globule forms of the protein that need to be rapidly cleared by the proteasome to prevent their accumulation and toxicity.

In the absence of ubiquitin or in addition to ubiquitin, high affinity interaction like those mediated by sequences within the protein like the predicted A helix region may be important in preventing premature release of a substrate. Similar to what seems to be happening with apoMb, ATPase induced unfolding by the mechanism of helix unraveling is likely to create intermediates in other proteins. Such unfolding intermediates in single domain proteins would mimic multi domain proteins in which the degradation signal meets up with a difficult domain or leads into a beta strand that seems resistant to unfolding by the proteasome [17], [18]. Therefore in addition to the structure adjacent to the degradation signals, the intermediates formed in globular proteins regardless of their secondary structural status may be key determinants of the rate of degradation. Such intermediates probably are impediment to global unfolding of the substrate by the ATPases.

#### Origin of intrinsic degradation signal or native degron in proteins

It has been well demonstrated that even when a protein is ubiquitinated, efficient degradation requires long unstructured regions [17], [18]. However what is unclear is the origin of such unstructured regions in proteins. By using a substrate that can be degraded in the absence of extrinsic degradation tags. Our study identifies the possible origin of such intrinsic degradation signals. The minimal required length of the disordered sequence seems to be a short stretch that is 29 residues long in the middle of the protein which originates from a well-defined secondary structural element like the α-helix. This region remains buried and is exposed only upon ligand removal. Long disordered regions ≥100 residues are common among intrinsically disordered proteins. The source of short disordered segments within other folded proteins may be reminiscent of any one of the following. Short stretches of peptides lacking a well-defined electron density (mobile) have been identified in 40% of the proteins (all species) for which high resolution crystal structures are available in the Protein Data Bank (PDB). In addition sequences (2 residues and longer) that seem to be poised for structural transition to a disordered conformation called the ‘dual personality’ segments (some of them similar to the F-helix) have also been identified within the PDB [51], [52], [53]. Post translational modification like phosphorylation, ubiquitination or mutations (like L104C and L115C) may create such disordered segments [54], [55]. Such flexible segments may also be made available by conformational changes upon ligand removal or due to the release of an interacting partner.

In summary, we have unequivocally demonstrated that purified eukaryotic 26S proteasomes can degrade a folded protein *in vitro* by recognizing sequence/s present within the substrate unaided by ubiquitin or adaptor proteins. While confirming to the known mechanism of degradation of ubiquitinated multi domain proteins fused with unstructured regions, degradation of apoMb and its mutants has revealed several new insights. We believe that apoMb would serve as a new model protein for in depth characterization of the sequence, structure, thermodynamic and kinetic aspects of degradation and the role of proteasomal ATPases in facilitating degradation. In addition since apoMb can be directly recognized by the proteasome and degraded without ubiquitin, it would be an appropriate model protein to investigate the explicit role of ubiquitination in proteasomal degradation.

## Materials and Methods

### Plasmids

Sperm whale Mb cDNA was a gift from S.G.Sligar [33]. Mutations in Mb were created by site directed mutagenesis (primers– **Table S4**) and sequence verified.

### Proteins

Myoglobin and its mutants were purified by cation exchange chromatography using CM52 cellulose (Whatman). Mb over expressing DH5α cells were lysed in 10 mM PO_4_ buffer (pH 6.8) by sonication. The pH of lysate was adjusted to 6.4 and incubated on ice for 1 h. After centrifugation the lysate was loaded on to CM cellulose column. After thorough washing, bound Mb was eluted with 30 mM PO_4_, pH 6.8. Purity was checked by 15% SDS-PAGE. ApoMb was prepared by acid-acetone method [34]. The heme free proteins were extensively dialyzed against milliQ water at 4°C. Heme removal was confirmed by recording the UV-visible spectrum between 240 to 500 nm (Jasco v-650). Protein concentrations (in milliQ water) were determined by UV absorbance at 280 nm using an extinction coefficient 15470 M^−1^ cm^−1^ for ApoMb (as estimated by ExPASy-ProtParam tool which agrees well with literature reported value [42]) and 34500 M^−1^ cm^−1^ for holoMb [42]. Proteins with A_280/260_ ratio ≥1.5 were used for all assays. After completion of the experiment (CD), proteins were analyzed by SDS-PAGE to further ensure that equivalent concentrations of different proteins were indeed used.

To check the quaternary structure of apoMb gel filtration was performed using Superdex 75 (GE Healthcare) matrix on Biologic duo flow (Bio-rad). A_280_ of the holo and apoMb loaded on the column were the same.

Yeast strains carrying the flag tagged 26S and 20S proteasome (YYS40-RPN11 3X FLAG) and YYS37-PRE1 3XFLAG; kind gift by Hideyoshi Yokosawa) were purified by affinity chromatography [35].

### Proteasomal degradation

12 µM substrate and 50 nM 26S or 20S proteasomes were incubated in the degradation buffer (20 mM HEPES/NaOH pH 7.5, 3 mM ATP, 15 mM MgCl_2_, 1 mM DTT, 2.5–3.5% glycerol). Substrate alone or substrate with MG132 treated 26S proteasome was taken as control. Degradation was also tested in the presence of ATPγS a non-hydrolysable analogue of ATP. The nucleotides obtained from Sigma were used directly. In incubations with ATPγS (3 mM), the final assay buffer contained 0.3 mM ATP from the elution buffer used to purify proteasomes. Other active site inhibitors of proteasome Velcade and epoxomicin as well as the serine protease inhibitor PMSF were tested likewise.

All reactions were carried out at 37°C. 10 µl aliquots were withdrawn at 0 h, 8 h, 12 h, 16 h and 19 h and added to 3X-SDS sample loading buffer, and stored at −20°C. These aliquots were resolved on a 15% SDS-PAGE. After Coomassie brilliant blue staining, substrate remaining was quantified by Image-J.

### Proteasome-substrate interaction

Interaction of proteasome with the substrate was monitored by ELISA. Briefly Nunc Maxisorb plate was coated with 2 µg/ml of anti-FLAG antibody (Sigma) in 100 mM sodium carbonate buffer pH 9.5. Plate was blocked with TBST containing 2% BSA. 1 µg/ml (0.37 nM) 26S proteasome was then captured using the dilution buffer (TBST supplemented with 1 mM ATP, 5 mM MgCl_2_ and 0.1%BSA). After washing with TBST, varying concentrations of apoMb (580 nM to 0.006 nM) were incubated with immobilized proteasomes. Finally, the amount of proteasome bound Mb was quantified by antiMb antibody (1∶500, Cell Signaling) followed by antimouse HRP antibody (1∶5000, Amersham) with TMB as the substrate. The reaction was stopped using 2 M H_2_SO_4_, and absorbance was measured at 450 nm (Spectra max190). Kd was calculated by fitting the data, using Graph Pad Prism 5 assuming one site specific binding (the two 19S caps were considered to be equivalent).

To detect binding of myoglobin derived peptides to the proteasome ELISA was similarly performed with biotinylated peptides (10 µM to 0.1 µM). Bound peptides were detected using streptavidin alkaline phosphatase and quantitated using 100 µl of 1× pNPP substrate. After 20 minutes the reaction was stopped with of 2N NaOH and absorbance was measured at 405 nm.

Ability of peptides to compete with Mb for binding to the proteasome was tested by incubating 8 nM apoMb in presence of varying concentrations (100 µM to 0.01 µM) of the peptides. Ki was calculated by fitting the graph assuming one site binding.

### ATPase assay

To characterize the purified proteasome and to establish the ability of proteasome to recognize apoMb, ATP hydrolysis was monitored in the presence and the absence of the substrate. Assay buffer (25 mM HEPES/NaOH pH7.5, 3 mM ATP,15 mM MgCl_2_) containing 0.01 µg/µl 26S proteasome (3.7pM) with or without 5 µg (580 nM) of the substrate was incubated at 37°C for 15 min. Amount of inorganic phosphate formed was estimated by calorimetry [56] (Spectra max190) and quantified using a standard graph.

### Limited proteolysis

Limited proteolysis of apowt or apoF-helix was performed using trypsin or chymotrypsin at 1/25 (w/w) ratio of enzyme to substrate in 20 mM Tris pH7.5 (1 mM CaCl_2_ was supplemented in case of chymotrypsin). Aliquots were withdrawn at 0, 15, 30 and 60 min and the reaction was stopped by adding 3X-SDS sample buffer for Tricine-PAGE. These aliquots were resolved on Tricine SDS-PAGE and stained by Coomassie brilliant blue.

### Far-UV Circular Dichroism

Far-UV CD spectrum (Jasco, J815) of Mb and its mutant proteins was recorded between 260 and 190 nm (Settings: Scan speed 50 nm/sec, accumulation 5, data pitch 0.1, at 24°C) in 20 mM PO_4_ buffer, pH 7.5. In case of cysteine mutants of Mb samples were first treated with 1 mM DTT in 20 mM PO4 buffer, pH 7.5 for 1 h at 37°C. All data were converted to mean residue ellipticity. The data were saved in Dichroweb format and subsequently analyzed by SELCON3 and CONTIN using Dichroweb [57], [58]. Thermal denaturation of wt Mb and its mutants was followed by scanning the CD spectra from 10°C to 90°C with an increment of 1°C/min. Sample was equilibrated for 5 min at each given temperature. Ellipticity at 222 nm at different temperature was used to calculate fraction unfolded. To determine the thermodynamic stability of wtapoMb and the F-helix mutant, both proteins (5 µM) were incubated overnight in different concentrations of urea (PO_4_ buffer pH 7.5) at 25°C and the CD spectra were taken. The data was fitted as described by Shirley [44].

### Tryptophan fluorescence

Trp environment in wtapoMb and mutant proteins was analyzed by fluorescence spectroscopy. Excitation was set at 295 nm and emission was monitored between 305–400 nm (slit width 5 nm, Fluorolog, Horiba). Fluorescence intensity of protein was subtracted from the buffer and the data is represented as relative fluorescence intensity.

## Supporting Information

Figure S1 **Characterization of affinity purified proteasome.** (a) To ensure that the purified proteasomes are intact and active, 20S and 26S proteasomes were resolved on a 4% native PAGE. In gel peptidase activity was performed by incubating the gels with Suc-LLVY-AMC and for the detection of 20S proteasome activity, 0.05% SDS was also used. Gels were stained with Coomassie brilliant blue to detect the proteins. (b) Subunit composition was verified by resolving purified 26S proteasome on a 12% SDS-PAGE.
(TIF)

Figure S2 **Stability of 26S proteasome and apoMb.** (A) Two different preparation (P1 and P2) of 26S proteasome was incubated at 37°C for 12 h in assay buffer. In gel activity and coommassie staining of native gel was performed. (B) Wt apoMb was incubated at 37°C and at the indicated time CD and fluorescence spectra were collected.
(TIF)

Figure S3 **Both apoMb and holo form bind with 20S proteasomes.** ApoMb and holoMb were incubated with immobilized 20S proteasome and detected using anti-Mb antibody.
(TIF)

Figure S4 **F-helix stabilization does not significantly affect the thermal stability or the Trp environment of Mb.** (a) Thermal denaturation of apo wt and F-helix mutant was monitored by secondary structural changes. Ellipticity at 222 nm was used to calculate the fraction folded which is plotted against the incubation temperature. (b) Trp fluorescence of wt apoMb and the F-helix mutant was analyzed under native conditions. Trp environment, an indicator of tertiary fold was similar in the wt and mutant proteins.
(TIF)

Figure S5 **Identification of proteasome interacting region/s on apoMb by peptide panning.** (a) Overlapping, biotinylated peptides (1 µM) corresponding to the primary sequence of Mb were incubated with the immobilized proteasome. A-helix peptide bound tightly to the proteasome, while B-helix, CD-loop and F-helix peptide bind weakly. (b) Amino acid sequences of peptides which were used for competition experiments are listed.
(TIF)

Figure S6 **Proteolytic stability of leu mutants of Mb.** (A) Limited proteolysis of L104C and L115C mutant was done with chymotrypsin, F-helix in these proteins seems to be more unstructured than wt. (B) Relatively less structured L104C protein was incubated with 20S proteasome, substrate remaining was quantified as described in methods, L104C was stable for degradation while an unstructured protein casein was degraded by 20S proteasome.
(TIF)

Table S1 Model systems used to study the mechanism of degradation of the eukaryotic proteasome.
(DOCX)

Table S2 Probing the stability of 26S proteasomes by Suc-LLVY-Amc cleaving activity.
(DOCX)

Table S3 Conformational Stability of wt and mutant ApoMb.
(DOCX)

Table S4 Primer sequences used for site directed mutagenesis of Mb.
(DOCX)

Method S1 Molecular dynamic simulation of wt myoglobin and F-helix mutant.
(DOCX)
