# Supplementary figures and images for: Direct Ubiquitin Independent Recognition and Degradation of a Folded Protein by the Eukaryotic Proteasomes-Origin of Intrinsic Degradation Signals
Source: PLoS One. 2012 Apr 10;7(4):e34864. doi: 10.1371/journal.pone.0034864 (PMC3323579; doi:10.1371/journal.pone.0034864)

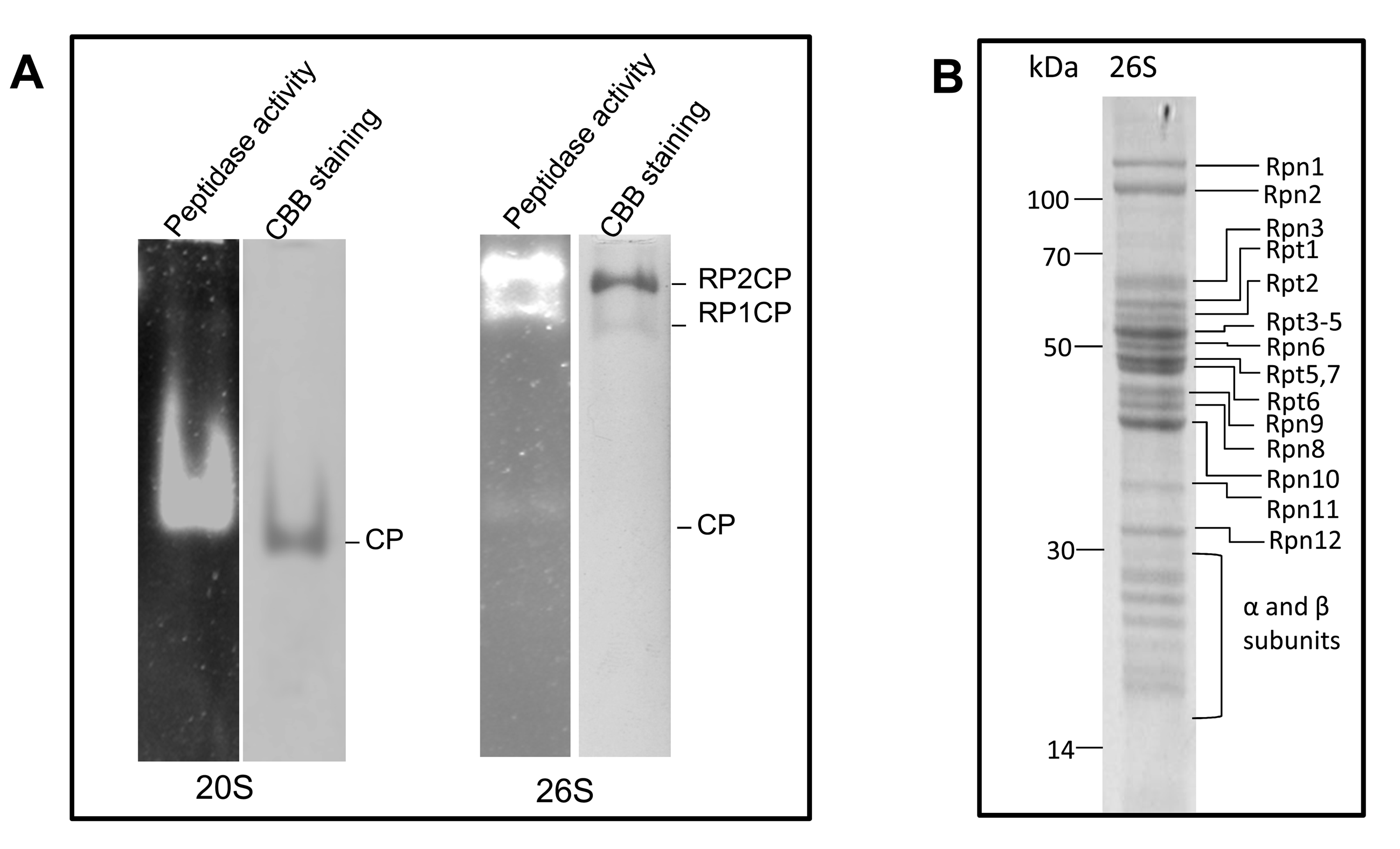

Supplement: Figure S1 — Characterization of affinity purified proteasome. (a) To ensure that the purified proteasomes are intact and active, 20S and 26S proteasomes were resolved on a 4% native PAGE. In gel peptidase activity was performed by incubating the gels with Suc-LLVY-AMC and for the detection of 20S proteasome activity, 0.05% SDS was also used. Gels were stained with Coomassie brilliant blue to detect the proteins. (b) Subunit composition was verified by resolving purified 26S proteasome on a 12% SDS-PAGE. (TIF) [file pone.0034864.s001.tif]

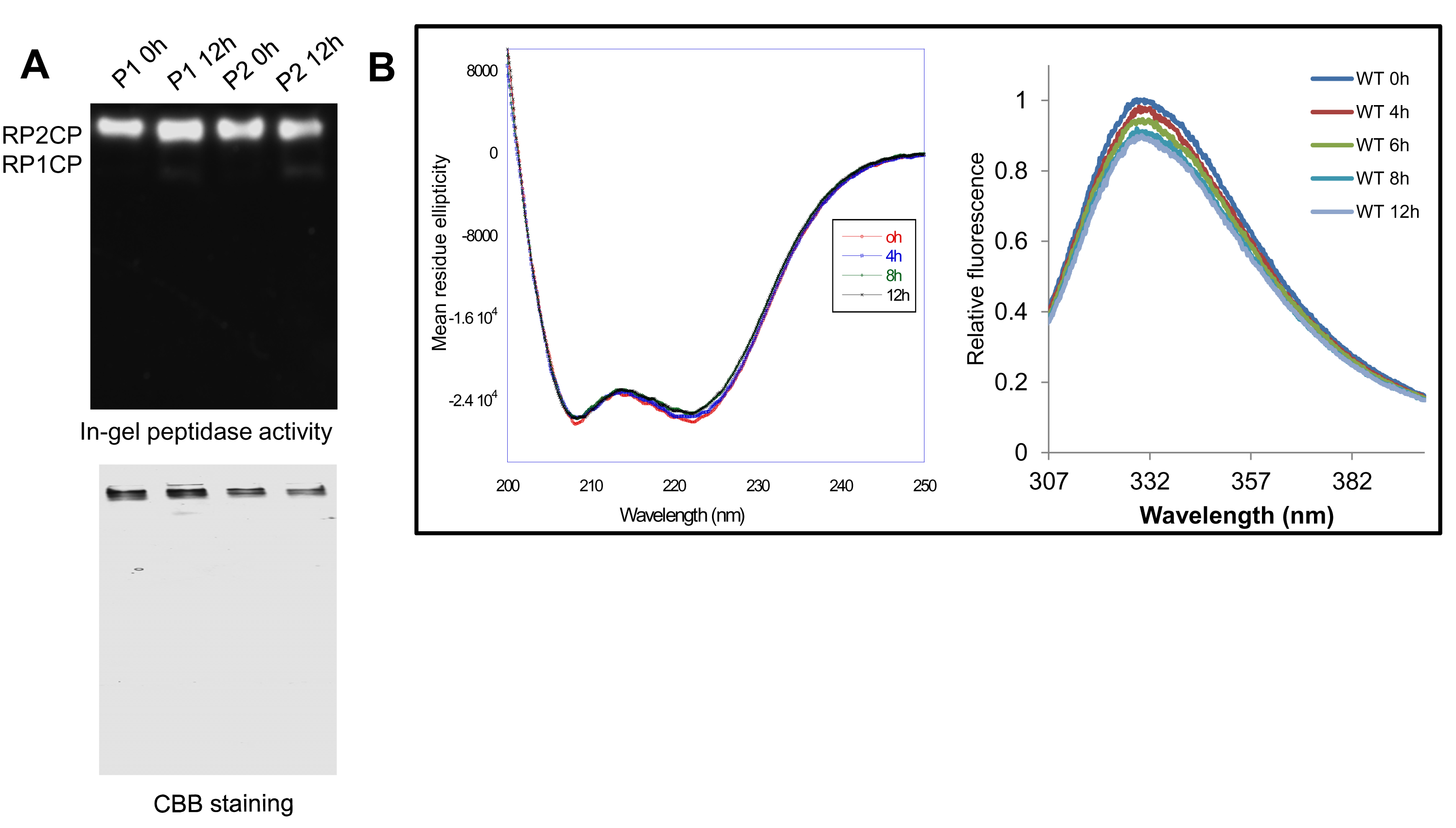

Supplement: Figure S2 — Stability of 26S proteasome and apoMb. (A) Two different preparation (P1 and P2) of 26S proteasome was incubated at 37°C for 12 h in assay buffer. In gel activity and coommassie staining of native gel was performed. (B) Wt apoMb was incubated at 37°C and at the indicated time CD and fluorescence spectra were collected. (TIF) [file pone.0034864.s002.tif]

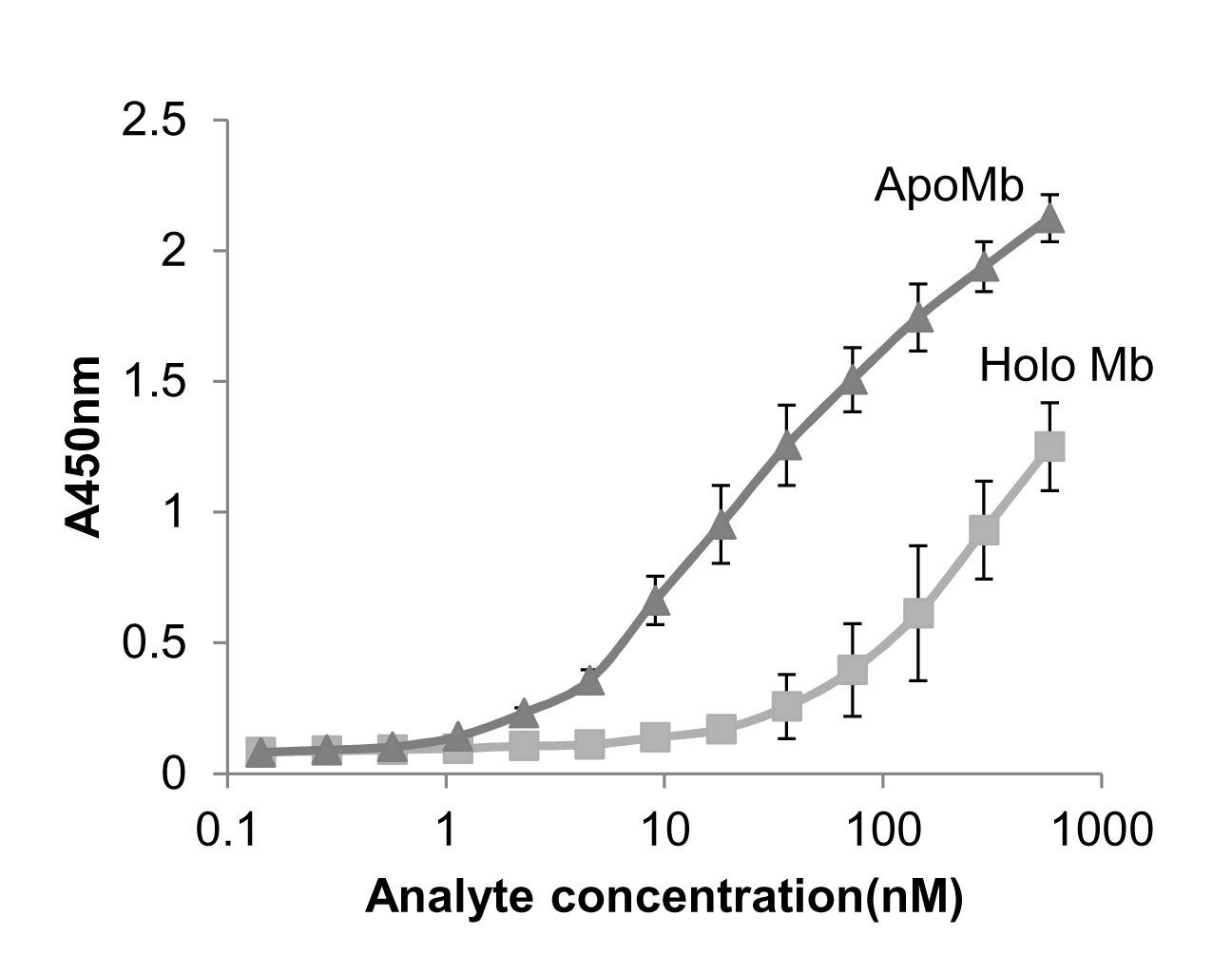

Supplement: Figure S3 — Both apoMb and holo form bind with 20S proteasomes. ApoMb and holoMb were incubated with immobilized 20S proteasome and detected using anti-Mb antibody. (TIF) [file pone.0034864.s003.tif]

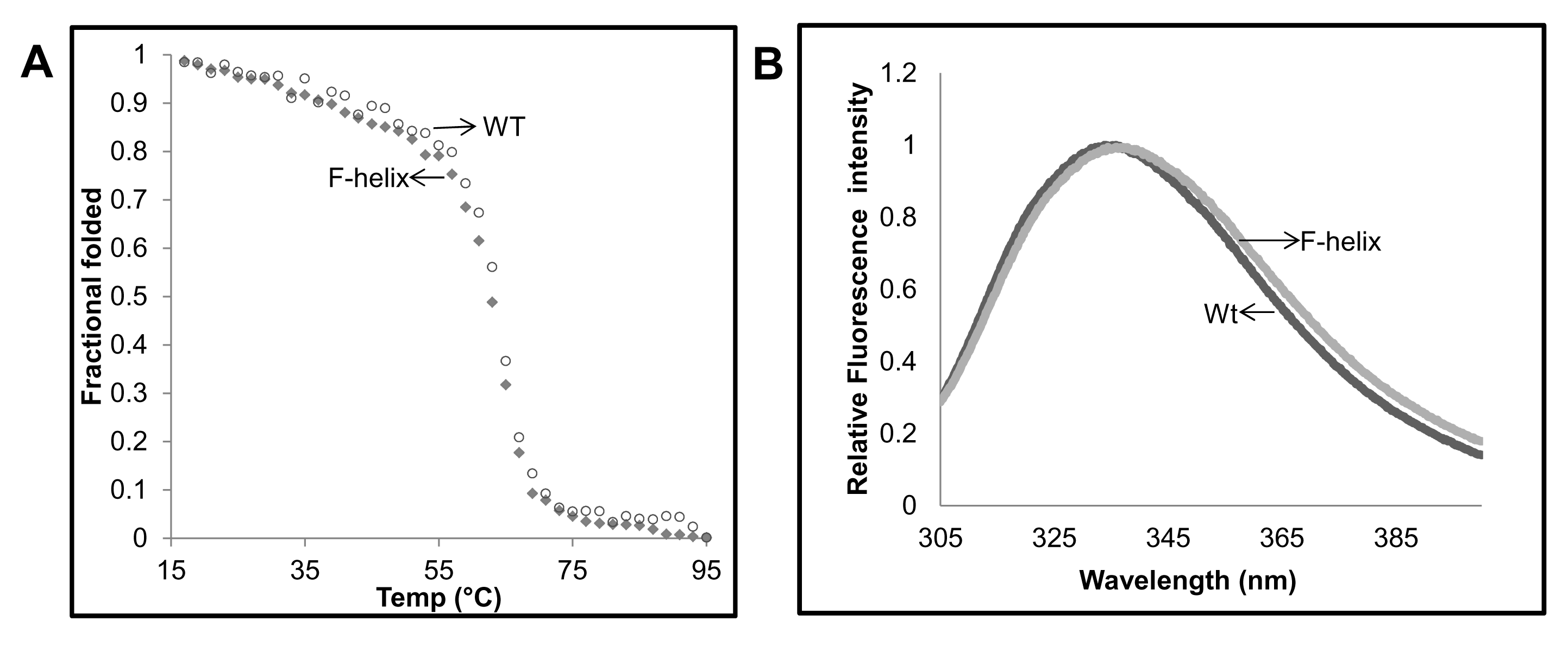

Supplement: Figure S4 — F-helix stabilization does not significantly affect the thermal stability or the Trp environment of Mb. (a) Thermal denaturation of apo wt and F-helix mutant was monitored by secondary structural changes. Ellipticity at 222 nm was used to calculate the fraction folded which is plotted against the incubation temperature. (b) Trp fluorescence of wt apoMb and the F-helix mutant was analyzed under native conditions. Trp environment, an indicator of tertiary fold was similar in the wt and mutant proteins. (TIF) [file pone.0034864.s004.tif]

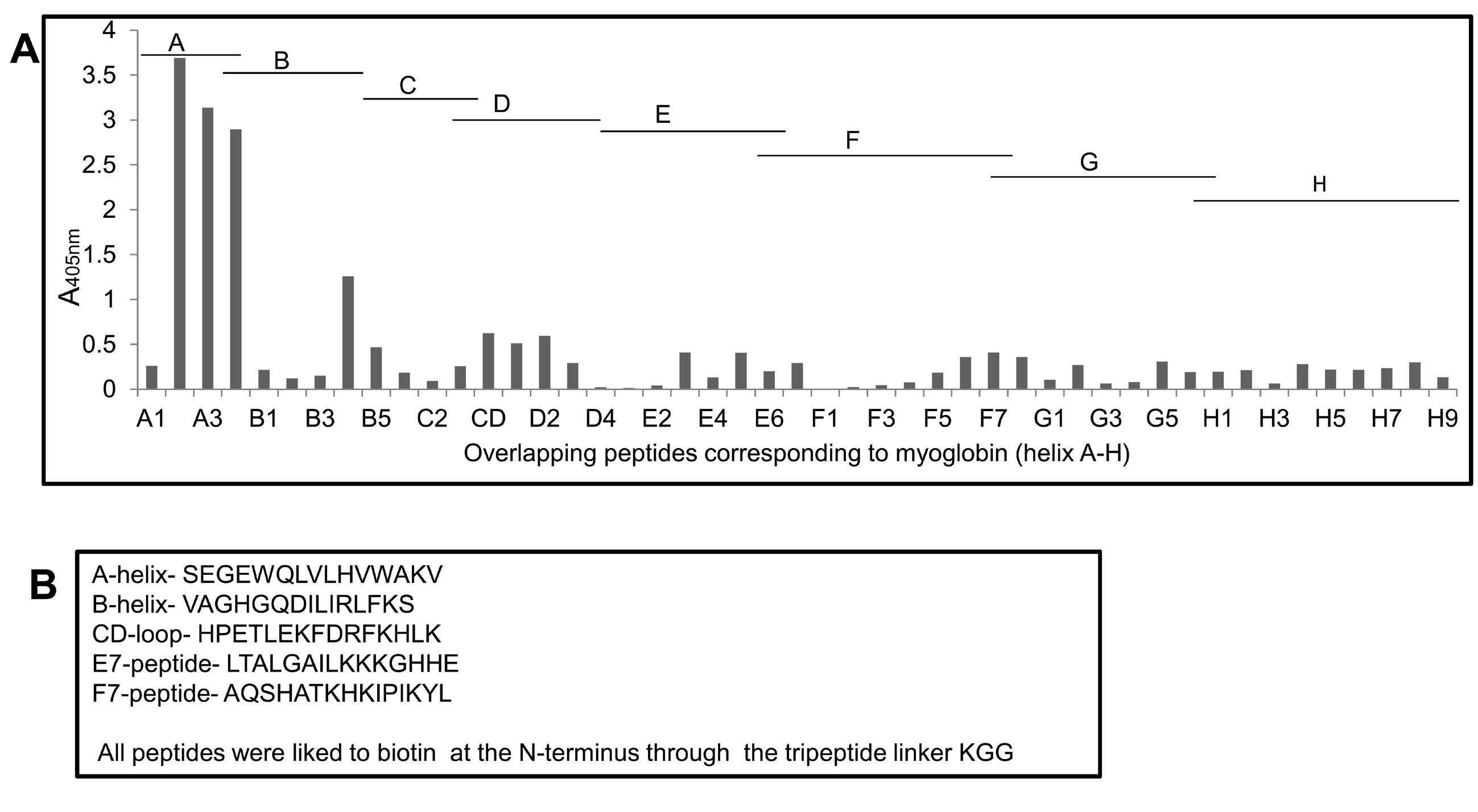

Supplement: Figure S5 — Identification of proteasome interacting region/s on apoMb by peptide panning. (a) Overlapping, biotinylated peptides (1 µM) corresponding to the primary sequence of Mb were incubated with the immobilized proteasome. A-helix peptide bound tightly to the proteasome, while B-helix, CD-loop and F-helix peptide bind weakly. (b) Amino acid sequences of peptides which were used for competition experiments are listed. (TIF) [file pone.0034864.s005.tif]

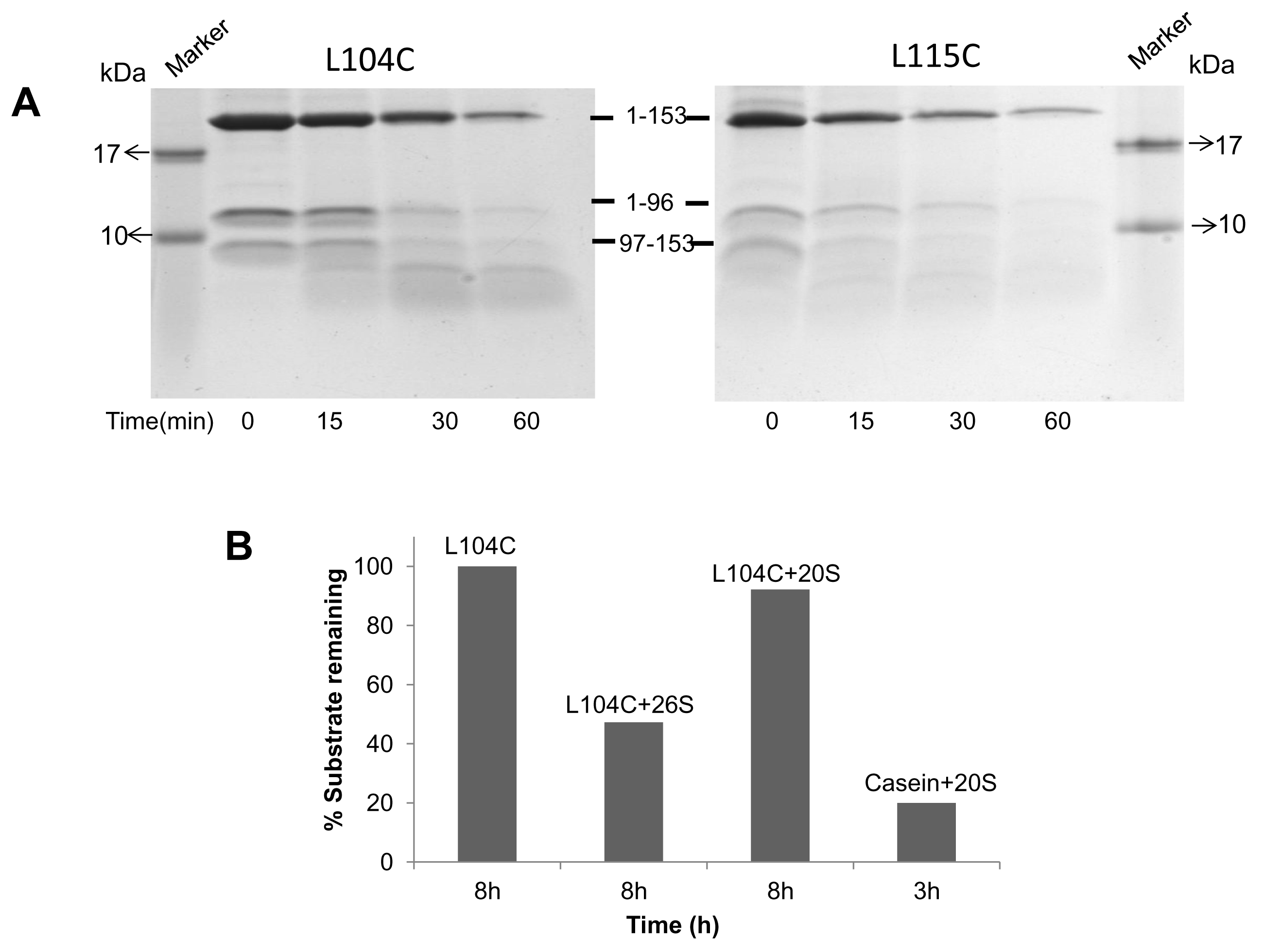

Supplement: Figure S6 — Proteolytic stability of leu mutants of Mb. (A) Limited proteolysis of L104C and L115C mutant was done with chymotrypsin, F-helix in these proteins seems to be more unstructured than wt. (B) Relatively less structured L104C protein was incubated with 20S proteasome, substrate remaining was quantified as described in methods, L104C was stable for degradation while an unstructured protein casein was degraded by 20S proteasome. (TIF) [file pone.0034864.s006.tif]
