# Supplementary material for: Direct Ubiquitin Independent Recognition and Degradation of a Folded Protein by the Eukaryotic Proteasomes-Origin of Intrinsic Degradation Signals
Source: PLoS One. 2012 Apr 10;7(4):e34864. doi: 10.1371/journal.pone.0034864 (PMC3323579; doi:10.1371/journal.pone.0034864)
Supplement: Table S1 — Model systems used to study the mechanism of degradation of the eukaryotic proteasome. (DOCX) [file pone.0034864.s007.docx]

**Supplementary Table S1.**

|  | Substrate | Source of  substrate | Source of proteasome | ubiquitination | Degradation signal /Unstructured extension used |
| --- | --- | --- | --- | --- | --- |
| Sumit Prakash *et al;* Nature Chemical Biology,Vol 5,number 1,2009  **Matouschek group** | N-degron DHFR-barstar hybrid | *In vitro* translation | Reticulocyte lysate | N terminal ubi,  N end rule pathway | Linker and 18 residue (318-335) from lac repressor |
|  | N-degron-DHFR-barstar and USR barnase heterodimer | *In vitro* translation | Reticulocyte lysate | N terminus DHFR-barstar is ubiquitinated | USR-Unstructured region derived from lac repressor or cytochrome b2 |
|  | Ub4-USR2-barstar and USR1 barnase | Purified | Purified proteasome | Ub tag is attached **102 amino acid** long linker or 129 amino acid p105 | URS 1- one copy of unstructured region. USR2- **210 amino acid** long, 2 copies of **1-95** residues of cytochrome b2 |
| Cheolju Lee *et al;* Molecular Cell, Vol. 7, 627–637, March, 2001  **Matouschek group** | DHFR, circularly permutated DHFR, Barnase, DHFR-barnase fusion | *In vitro*  translation | Reticulocyte lysate | Ubi is attached to 40aminoacid linker derived from lac repressor | Lac repressor |
| Sumit prakash et al; Nature Molecular and Structural Biology, Vol 11,Number 9, 2004  **Matouschek group** | N-Usignal-DHFR-barnase-C, N-barnase-DHFR-Usignal-C | *In vitro*  translation  and partial purification by ammonium sulphate | Reticulocyte lysate | N-end rule pathway | **1–95 of cytochrome *b*2 or**  **residues 1–40 of lac repressor** |
|  | N-Usignal-DHFR-unstructured-6Xhis | Purified | Reticulocyte lysate | ubi was attached N to C terminus | 1–95 of cytochrome *b*2 or  residues 1–40 of lac repressor |
| Susan Fishbain *et al;* Nature communication,  8 Feb 2011, DOI: 10.1038/ncomms1194  **Matouschek group** | N-UBL- DHFR-RBD-DHFR-C, N-UBL-UBA-RBD-UBA-unstructured region-C and its different combinations | *In vitro*  translation  and partial purification by Ammonium Sulphate | Purified  They have also done in vivo | UBL and 2 UBA domain | 1–95 of cytochrome *b*2 or  residues 1–40 of lac repressor |
| Minglian Zhao *et al;* JBC, VOL. 285, Number 7, 2010. | UbcH10,Ub4 UbcH10, Ub4 PEST UbcH10 | Purified | Purified | Tetra ubi was used | **PEST- 37aa of ODC** |
| Allen Henderson *et al*; JBC, VOL. 286, Number 20, 2011. | Titin I27-cODC,RPN10- Titin I27-cODC,RPN10-DHFR-cODC | Purified | Purified |  | RPN10 was used for localization and cODC as PEST |
| Chang-Wei Liu, *et al; Science* 299, 408, 2003 | p21,α-synuclein | Purified | 20S and 26S Purified |  | Both proteins are natively disordered |
|  | GFP-p21, GFP- α-synuclein, GFP-p21-GFP and GFP- α-synuclein-GFP | Purified | 20S and 26S Purified | ubi not required | p21,α-synuclein proteins are natively disordered |
| From **Coffino group-** Zhang *et al*; EMBO J. 22, 2003.  Takeuchi *et al*; EMBO J. 26 2007.  Takeuchi *et al*; Biochem. J. 410, 2008. | Ornithine decarboxylase (ODC),  DHFR ODC, GFP ODC | Purified radio labeled | Purified and reticulocyte lysate | ubi not required.  Antizyme 1 (AZ1) is mandatory for ODC degradation | disordered  37-residue region at the C-terminal end, C 441 is must |
| Daniel M. Janse *et al;* JBC, Vol. 279, No. 20, 2004 | Fpr1-rapamycin  binding domain of Tor1 ,  proteasome subunit Fpr1 complex, Tor fused His 3 | Purified | Purified | ubi not required.  Fpr1 was fused to proteasomal subunit. Tor was fused to His3 | No external disorder fusion, Addition for rapamycin heterodimerize Fpr1 and tor1 and His3 translocate to proteasome |
| Antonia M. Forsthoefel et al; Biochemistry 43, 2004. | Thymidylate synthase | In vivo (cycloheximide treatment ) | Cellular | Ubi independent | Internal N-terminal, |
| James M. Baugh et al; *J Mol Biol*, 386(3), 2009. | GST-HR23A (they have also shown 30proteins from cell lysate to be degraded by 20S) | Purified | Purified 26S and 20S | Ubi independent | Degradation with cleaved products formation |
