# Supplementary material for: Direct Ubiquitin Independent Recognition and Degradation of a Folded Protein by the Eukaryotic Proteasomes-Origin of Intrinsic Degradation Signals
Source: PLoS One. 2012 Apr 10;7(4):e34864. doi: 10.1371/journal.pone.0034864 (PMC3323579; doi:10.1371/journal.pone.0034864)
Supplement: Table S2 — Probing the stability of 26S proteasomes by Suc-LLVY-Amc cleaving activity. (DOCX) [file pone.0034864.s008.docx]

**Supplementary Table S2.** Probing the stability of 26S proteasomes by Suc-LLVY-Amc cleaving activity

|  | P1 | P2 |
| --- | --- | --- |
| 0h | 100% | 100% |
| 12h | 88% | 87% |

Activity was monitored in the 26S assay buffer in which the 20S proteasome is inactive.P1 and P2 are two different proteasome preparations.
