# Supplementary material for: Direct Ubiquitin Independent Recognition and Degradation of a Folded Protein by the Eukaryotic Proteasomes-Origin of Intrinsic Degradation Signals
Source: PLoS One. 2012 Apr 10;7(4):e34864. doi: 10.1371/journal.pone.0034864 (PMC3323579; doi:10.1371/journal.pone.0034864)
Supplement: Table S3 — Conformational Stability of wt and mutant ApoMb. (DOCX) [file pone.0034864.s009.docx]

**Supplementary Table S3.** Conformational Stability of wt and mutant ApoMb

|  | Relative circular dichorism (222nm) | | | | Relative tryptophan fluorescence | | | |
| --- | --- | --- | --- | --- | --- | --- | --- | --- |
|  | 0h | 4h | 8h | 12h | 0h | 4h | 8h | 12h |
| WT | 1 | 0.98 | 0.95 | 0.97 | 1 | 0.96 | 0.91 | 0.89 |
| F-HELIX | 1 | 1 | 0.98 | 0.98 | 1 | 0.99 | 0.98 | 0.93 |
| L104C | 1 | 1 | 0.95 | 0.95 | 1 | 1 | 0.99 | 0.97 |
| L115C | 1 | 0.93 | 0.93 | 0.92 | 1 | 1 | 0.99 | 0.89 |
