# Supplementary material for: Direct Ubiquitin Independent Recognition and Degradation of a Folded Protein by the Eukaryotic Proteasomes-Origin of Intrinsic Degradation Signals
Source: PLoS One. 2012 Apr 10;7(4):e34864. doi: 10.1371/journal.pone.0034864 (PMC3323579; doi:10.1371/journal.pone.0034864)
Supplement: Table S4 — Primer sequences used for site directed mutagenesis of Mb. (DOCX) [file pone.0034864.s010.docx]

**Supplementary Table S4.** Primer sequences used for site directed mutagenesis of Mb.

| **Mb mutant** | **Primes** |
| --- | --- |
| F-helIx1F (P88AS92AH97N) | CTCAAAGCGCTTGCGCAAGCGCATGCTACTAAAAACAAGATCC |
| F helIx1R (P88AS92AH97N) | GGATCTTGTTTTTAGTAGCATGCGCTTGCGCAAGCGCTTTGAG |
| F helex 2 F (G80A) | CCTTAAGAAAAAAGCCCATCATGAAG |
| F helex 2 R (G80A) | CTTCATGATGGGCTTTTTTCTTAAGG |
| V10C F | GAATGGCAGCTGTGCCTGCATGTTTGGGC |
| V10C R | GCCCAAACATGCAGGCACAGCTGCCATTC |
| T39C F | CTCATCCGGAATGCCTGGAAAAATTCGATCG |
| T39C R | CGATCGAATTTTTCCAGGCATTCCGGATGAG |
| L104C F | AGAGATGAATTCGCAGTATTTGATCGG |
| L104C R | CCGATCAAATACTGCGAATTCATCTCT |
| L115C F | CGATCATCCATGTTTGCCATTCTAGACATCC |
| L115C R | GGATGTCTAGAATGGCAAACATGGATGATCG |
| M131C F | CTCAGGGTGCTTGCAACAAAGCTCTCGAG |
| M131C R | CTCGAGAGCTTTGTTGCAAGCACCCTGAG |
