# Supplementary material for: Direct Ubiquitin Independent Recognition and Degradation of a Folded Protein by the Eukaryotic Proteasomes-Origin of Intrinsic Degradation Signals
Source: PLoS One. 2012 Apr 10;7(4):e34864. doi: 10.1371/journal.pone.0034864 (PMC3323579; doi:10.1371/journal.pone.0034864)
Supplement: Method S1 — Molecular dynamic simulation of wt myoglobin and F-helix mutant. (DOCX) [file pone.0034864.s011.docx]

Molecular dynamic simulation was performed using AMBER 10 and AmberTools. The initial co-ordinates were obtained from the PDB file 2JHO. The holo sperm whale myoglobin was then stripped of all metal ions and the heme moiety to create apomyoglobin. All the hydrogen atoms and anisotropic atoms were removed. A program from Amber tools called LEaP was then used to add the hydrogen atoms. ApoMb was solvated in TIP3P model of water. For energy minimization using Sander, a combination of steepest descent and conjugate gradient methods was used for 2000 steps. Energy minimized ApoMb was equilibrated to a temperature of 400K over 10 ps. An all atom force field was used for simulation with the temperature set to 400K for a period of 2.8 ns. The trajectories resulting from the simulation were converted to a PDB file and analyzed using Visual Molecular Dynamics (VMD).The apomyoglobin was then mutated with residues intended to stabilize the F-helix (indicated below). The mutant was created and modeled using modeler 9v7 with 2JHOas the template. Molecular dynamic simulation of the mutant was performed exactly as described for the wild type

- Original sequence of F-helix

K (G) H H E A E L K (P) L A Q (S) H A T K (H) K I P

- Mutated sequence

K (A) H H E A E L K (A) L A Q (A) H A T K (E) K I P
